# Multimodal pre-training models of molecular representation for drug discovery

**DOI:** 10.1093/nsr/nwaf495

**Published:** 2025-11-11

**Authors:** Xiaoqi Wang, Chuanshi Wang, Boya Ji, Junwen Wang, Mingyue Zheng, Lingyun Song, Shaoliang Peng, Xuequn Shang

**Affiliations:** School of Computer Science, Northwestern Polytechnical University, Xi’an, 710129, China; Key Laboratory of Big Data Storage and Management, Ministry of Industry and Information Technology, Northwestern Polytechnical University, Xi’an, 710129, China; School of Computer Science, Northwestern Polytechnical University, Xi’an, 710129, China; Key Laboratory of Big Data Storage and Management, Ministry of Industry and Information Technology, Northwestern Polytechnical University, Xi’an, 710129, China; College of Computer Science and Electronic Engineering, Hunan University, Changsha, 410082, China; Division of Applied Oral Sciences & Community Dental Care, Faculty of Dentistry, the University of Hong Kong, Hong Kong 999077, China; Drug Discovery and Design Center, State Key Laboratory of Drug Research, Shanghai Institute of Materia Medica, Chinese Academy of Sciences, Shanghai 201203, China; School of Computer Science, Northwestern Polytechnical University, Xi’an, 710129, China; Key Laboratory of Big Data Storage and Management, Ministry of Industry and Information Technology, Northwestern Polytechnical University, Xi’an, 710129, China; College of Computer Science and Electronic Engineering, Hunan University, Changsha, 410082, China; School of Computer Science, Northwestern Polytechnical University, Xi’an, 710129, China; Key Laboratory of Big Data Storage and Management, Ministry of Industry and Information Technology, Northwestern Polytechnical University, Xi’an, 710129, China

**Keywords:** self-supervised learning, multimodal pre-training model, Transformer, molecular representation, drug discovery

## Abstract

With the great success of large language models in natural language processing, self-supervised pre-training models have emerged as an important technique in drug discovery. In particular, multimodal pre-training models have opened a new avenue for drug discovery. The experience and ideas from previous works can provide important reference points for further research in drug discovery. Therefore, this review summarizes the foundation of multimodal pre-training models and their progress in the field of drug discovery. We emphasize the adaptability between various modalities and network frameworks or pre-training tasks. At the same time, we summarize the difference and relevance between various modalities or pre-training models. Importantly, we identify two increasing trends that may serve as reference points for future research. Specifically, Transformers and graph neural networks are often integrated as encoders and then combined with multiple pre-training tasks to learn cross-scale molecular representation, thereby promoting the accuracy of drug discovery. In addition, molecular captions as brief biomedical text provide a bridge for collaboration between drug discovery and large language models. Finally, we discuss the challenges of multimodal pre-training models in drug discovery, and explore future opportunities.

## Teaser

A systematic review summarizing the foundation of multimodal pre-training models and their progress in molecular representation of drug discovery.

## INTRODUCTION

**Figure 1.**
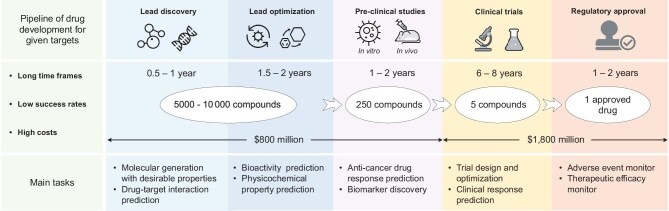
The key process of new drug development for a given target. Notably, this work aims to summarize multimodal pre-training models of molecular representation for drug discovery. However, pre-clinical studies, clinical trials and regulatory approval focus on the cellular or patient response rather than the molecular level. Therefore, this paper summarizes the tasks of drug discovery, including lead discovery and optimization.

Drug development is of great significance to human health. For given targets, the pipeline of new drug development includes lead discovery and optimization, pre-clinical studies, clinical trials and regulatory approval. This is an expensive and time-consuming process with a very low success rate [1,2]. It takes on average US$2.6 billion and 10–15 years to develop a new drug. Approximately, five of the screened 10 000 compounds in the initial stage are selected for clinical trials [3], as shown in Fig. 1. Unfortunately, the number of new drugs gaining regulatory approval per billion USD spent halves every nine years, a trend sometimes called ‘Moore’s law in reverse’ [4]. To improve efficiency of new drug development, deep-learning technologies driven by a great deal of biomedical data have been applied to the pharmaceutical industry [2,5,6]. In particular, AlphaFold [7,8], a deep-learning-based protein structure prediction method, has emerged as a key technology for drug research and development [9,10]. With the rapid development of deep learning, many advanced models, including convolutional neural networks [11,12], recurrent neural networks [13,14], graph neural networks [15–18] and Transformer [19], are used for multiple tasks in drug discovery. The optimization of these neural networks relies on the large-scale samples that are labeled with molecular information, such as biochemical properties, drug targets and side effects. However, labeled data are very rare in real drug discovery scenarios, limiting the performance and ability of deep learning [20]. Fortunately, self-supervised pre-training technologies have shown promise in reducing the negative effects of sparse data, thus bringing new opportunities for the further development of drug discovery.

Pre-training technologies have achieved remarkable success in the fields of natural language processing and computer vision. In particular, large language models have shown great capabilities in some applications [21,22]. In this context, pre-training technologies have also received increasing attention in drug discovery [23–25]. For example, UniCure [26] proposed a pre-training model for cancer therapy response prediction that helps to promote the development of drug discovery and personalized precision oncology. The key advantage of pre-training technologies is that models can learn potential patterns behind molecules via self-supervised tasks, in which supervision signals are automatically generated from unlabeled data [20,27]. Initially, inspired by BERT [28], the masked language model—a popular pre-training method—was applied to molecular sequences, such as the simplified molecular-input line-entry system (SMILES) [29]. The hypothesis behind this line of work is that molecular sequences can be treated as a type of scientific language [30,31]. Therefore, molecular sequences can directly interact with the masked language model to improve the performance of drug discovery. To capture higher-order structure and semantic features, there is increasing interest exploring pre-training strategies on two-dimensional (2D) molecular graphs, 3D geometries and molecular interaction networks [32–34]. At the same time, molecule captions, which refer to brief biomedical text descriptions, also provide a bridge between natural language processing and drug discovery. Therefore, numerous studies, such as MolT5 [35], and BioT5 [36], have designed pre-training models on a corpus composed of biomedical texts and molecule sequences for drug discovery. More recently, pre-training algorithms have moved from unimodal paradigms to multimodal paradigms. Research suggests that multimodal pre-training tasks are conducive to encouraging deep models to systematically understand the features of molecules, thus achieving the competitive results in drug discovery [34,37,38].

Multimodal pre-training models have emerged as a new paradigm in drug discovery and have shown great prospect. The experience and ideas within these previous works can provide the important reference points for the further development of drug discovery. Therefore, this paper presents a systematic review that summarizes the foundations and progress in the field of multimodal pre-training model-based drug discovery, in order to provide the reference points for future research. We summarize the foundations of molecular modalities and revisit the network frameworks, self-supervised tasks, training strategies and their applications in drug discovery. In each section, we emphasize the differences and relations between various modalities or methods. Simultaneously, we focus on the adaptability between various modalities and pre-training methods. Previous works suggest two increasing trends.

(1) There are increasing attempts to explore the combination of Transformer and graph neural networks to encode cross-scale molecular representations. Subsequently, these hybrid network frameworks are pre-trained by leveraging multiple self-supervised tasks, thus promoting the performance of drug discovery.

(2) Molecule captions, as brief biomedical texts, provide a bridge for collaboration between drug discovery and large language models.

Finally, we discuss the remaining challenges and explore future opportunities, including unified frameworks, discrimination of cross-modal consistency and complementarity, and fusion of biochemical knowledge with foundation models. We hope that this review will inspire future research to develop more advanced techniques for drug discovery. The key references investigated in this review can be accessed at https://github.com/AISciLab/MultiPM4Drug.git.

## MULTIMODAL PRE-TRAINING MODELS OF MOLECULAR REPRESENTATION

**Figure 2.**
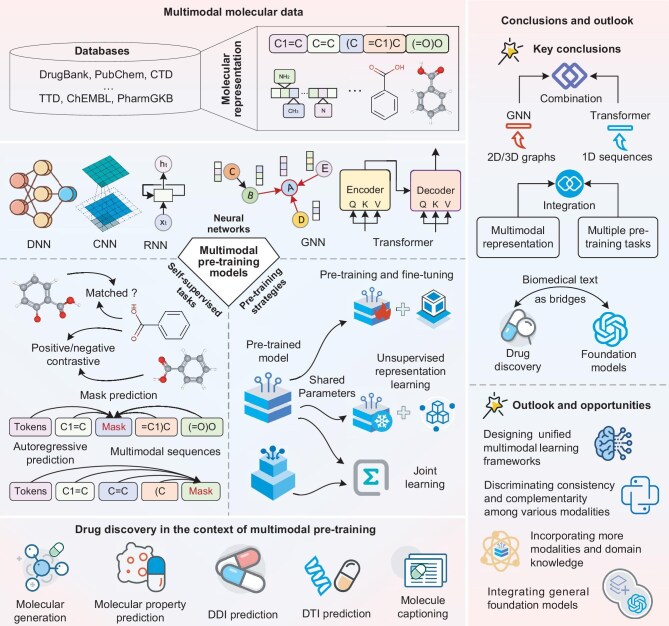
The key content and conclusion of this review.

We aim to summarize multimodal pre-training models of molecular representation for drug discovery, in which multimodal pre-training models refer to self-supervised tasks guiding the deep-learning networks to learn a unified representation for drug discovery. Notably, pseudo-labels of self-supervised tasks are automatically generated based on specific attributes of the multimodal data [39]. In multimodal pre-training models of molecular representation, there are four key components: molecular data, neural networks, pre-training tasks and pre-training strategies, as shown in Fig. 2. Specifically, a large amount of molecular data provides the opportunity for multimodal pre-training models. Subsequently, neural networks, acting as encoders, are pre-trained to encode molecular representations via self-supervised tasks. In cross-modal contrastive learning and matching prediction, there often are multiple embedding networks to encode different modalities, which are co-trained with cross-modal objectives. On the other hand, multiple types of input data are converted into the specific sequences [37,40], and then share the same encoder in cross-modal masked and autoregressive prediction. Subsequently, the pre-trained encoders are applied to drug discovery through different training strategies. More importantly, previous works suggest two increasing trends that may be used as reference points for future research.

(1) Transformers and graph neural networks are often integrated together as encoders, and then cooperate with multiple pre-training tasks to learn cross-modal molecular representation.

(2) Molecule captions, as brief biomedical texts, provide a bridge for collaboration between drug discovery and large language models.

Finally, we discuss the challenges of multimodal pre-training models in drug discovery and explore future opportunities.

## MULTIMODAL MOLECULAR DATA

**Figure 3.**
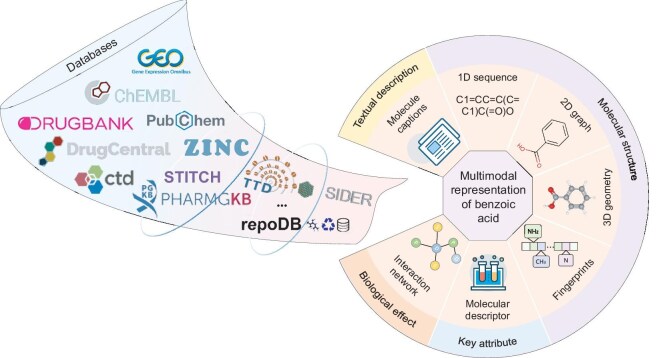
A diagram illustrating the multimodal representation of benzoic acid. Here, we summarize only part of the databases due to space limitations. More databases and their descriptions can been found in Section S1.

The growth of biomedical data provides vast opportunities for deep-learning-based drug discovery. The scale and quality of biomedical data are key factors for the successful application of deep learning in drug discovery. Therefore, a series of free and open-access databases with structured biomedical data has been developed to promote progress in drug discovery. In this work we aim to summarize multimodal pre-training models of molecular representation for drug discovery. Accordingly, we summarize the popular databases that focus on collecting the molecular information and provide a brief description in Section S1. In these databases, as shown in Fig. 3, molecules are encoded in different modalities, including molecular descriptors, 1D molecular sequences, 2D molecular graphs, 3D molecular structures, molecular interaction networks and textual captions (for a detailed description, see Section S1). These different modalities reflect the molecular features from different perspectives. The form of molecular modalities determines the framework and performance of deep learning for drug discovery.

For a given molecule, 1D sequences, 2D graphs, 3D geometries and molecular fingerprints describe its structure features at different scales. Compared to 1D sequences, 2D molecular graphs and 3D molecular geometries can capture the associations and spatial relationships between atoms, respectively. However, in most databases, molecules are usually represented via 1D sequences to reduce storage space. Different structure representations can be converted into one another using biochemical tools such as RDKit [41], and Open Babel [42]. Molecular descriptors reflect the physical and chemical properties of molecules. Molecular interaction networks represent the regulatory relationships among different biological entities in living systems. It is intuitive to hold that molecular interaction networks can simulate the natural law of ‘multiple drugs, multiple targets and multiple diseases’. In addition, drug interaction networks can integrate more information and are more suitable for the development of multi-omics fusion [43], compared with representations of other modalities. Compared with the representations of other modalities, molecule captions are more concise and intuitive. Unlike molecular structure and interaction networks, which require the reader to have some level of chemical knowledge, a molecule caption is often a concise overview of key characteristics. It is natural to regard a molecule caption as a textual description. Therefore, molecule captions are more suitable for large language models. However, for a given molecule, various organizations often employ different types of language for molecule captions, resulting in poor standardisation and uniformity. Based on the momentum of large language models, there is growing interest in jointly modeling molecular representations and natural language. In particular, multimodal fusion is beneficial for systematically understanding molecules and improves the robustness of deep learning [44,45].

## NEURAL NETWORK FRAMEWORKS

Multimodal representations of molecules are used to drive different neural networks for drug discovery, as shown in Table 1. These networks aim to learn complex relationships and high-quality representations from large volumes of data. The choice of network frameworks has a substantial impact on the predictive performance of drug discovery. Generally, five classical network architectures—deep neural networks (DNNs) [46], convolutional neural networks (CNNs) [11,12], recurrent neural networks (RNNs) [13,14], graph neural networks (GNNs) [15–17] and Transformer [19]—are widely applied to drug discovery.

**Table 1.** Analysis and comparison of different neural networks.

Model	Input data	Key modules	Characteristic
DNN	Structured vectors	Fully connected layer with nonlinear function	$\bullet$ Have simple network architectures
			$\bullet$ Ignore the relation among elements in data
CNN	Grid-like data	Convolutional and pooling operation	$\bullet$ Capture the local feature
			$\bullet$ Reduce the size of parameters
RNN	Sequence data	Recurrent units	$\bullet$ Capture temporal dependencies in data
			$\bullet$ Suffer from vanishing or exploding gradients for long sequences
Transformer	Long sequence data	Self-attention mechanisms, and positional encodings	$\bullet$ Capture long- and global-range dependencies
			$\bullet$ Suffer from higher computation complexity
GNN	Graph data	Spectral graph convolutions or message passing mechanisms	$\bullet$ Capture complex topology in graph data
			$\bullet$ Suffer from over-smoothing issues

These neural network frameworks show different levels of compatibility and adaptability for molecular representations with different modalities. The core modules of DNNs are fully connected layers with nonlinear functions (see Section S2). DNNs, as the basis of deep learning, have simple network architectures. However, DNNs ignore the interdependence among elements in input data. Therefore, DNNs are more suitable for structured vectors, such as molecular fingerprints and descriptors, in which each element denotes an independent feature value. By contrast, CNNs can capture the local structure in grid-like data, such as pixel distributions in images. In addition, CNNs reduce the size of parameters because the weights are shared in the convolutional kernels (see Section S3). Therefore, several studies hold that 2D molecular graphs can be treated as images and then fed into 2D CNNs for drug discovery [47–49]. On the other hand, numerous studies proposed 1D CNNs for sequential data by extending the principles of traditional convolutional networks [50]. In contrast to CNNs, RNNs are specifically designed for sequence data with temporal dependencies, such as textual descriptions, SMILES and audio. RNNs aim to learn the temporal dependencies in sequence data through recurrent units (see Section S4). Unfortunately, as sequence length increases, RNNs may suffer from vanishing or exploding gradient problems. To address this issue, Transformers employ a multi-head self-attention mechanism to calculate the relationships between different positions in sequential data, thus capturing long- and global-range dependencies. Simultaneously, Transformers integrate positional encodings to capture the order of each element in sequences.

Intuitively, DNNs, CNNs, RNNs and Transformers are designed for Euclidean-structured data but are difficult to apply to graph-structured data, such as social networks, transportation networks and protein-protein interaction networks. Therefore, GNNs have been developed to learn graph representations via spectral graph convolutions or message-passing mechanisms that can capture the topology of graph data [51]. Simultaneously, various geometric GNNs [52,53] and heterogeneous GNNs [54,55] extend the message-passing mechanism to geometric graphs and heterogeneous networks, respectively (see Section S5). However, deeper GNNs may suffer from over-smoothing issues, resulting in performance degradation [56]. Theoretically, different network frameworks have distinct characteristics. Inherently, these networks are modified or fused to improve performance in drug discovery.

**Figure 4.**
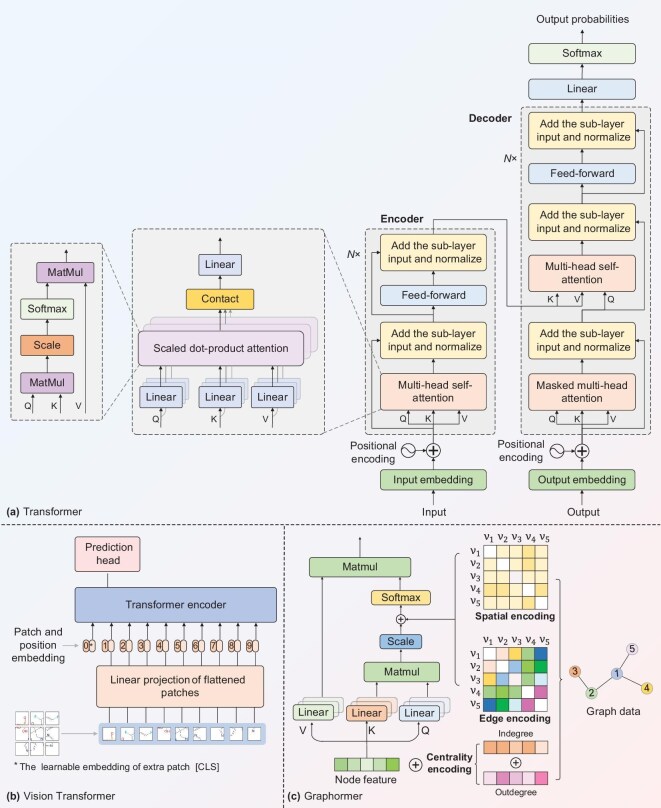
The architectures of different Transformer networks: (a) the original Transformer, (b) the original vision Transformer, (c) the Graphormer.

With the success of Transformers and GNNs on sequence and graph data, they have attracted increasing attention from the field of drug discovery [57]. In particular, the Transformer has emerged as a leading technique in multimodal pre-training models for drug discovery. The Transformer adopts an encoder-decoder framework as shown in Fig. 4a. The encoder maps sequential data into low-dimensional representations, while the decoder maps these representation vectors to specific outputs. A more detailed description of the Transformer can be found in Section S6.

*Encoder.* The encoder is composed of multiple modules with the identical structures. Each module contains a multi-head self-attention layer and a fully connected feed-forward network layer, which consists primarily of two linear layers with a ReLU non-linear function. Each sub-layer internally implements residual connections [58] and layer normalization [59] operations that can accelerate the convergence of the model. The self-attention mechanisms primarily learns the long-range dependencies among elements (e.g. tokens) in sequential data. [19] Within each module, multiple self-attention models are employed to increase stability.

*Decoder.* Similar to the encoder, the decoder is also composed of a stack of multiple identical modules, which mainly consist of multi-head attention layers and feed-forward network layers. In addition, the decoder introduces a masked-attention layer to prevent current positions from attending to subsequent positions. In other words, the masked attention ensures that the representation at each position depends only on information from the left of the current position. This is mainly because the output at each position only needs to consider the influence of known data to its left. Finally, the outputs are completed by a learnable linear transformation and Softmax function.

The core component of the Transformer is multi-head self-attention mechanisms, which aims to learn the long-range dependencies among elements (e.g. tokens) in sequential data. The Transformer has achieved a major breakthrough in drug discovery. Generally, SMILES sequences are treated as a special chemical language and then fed into the Transformer encoder to learn representations for drug discovery [60,61]. In drug-target interaction prediction and target-aware molecule generation, amino acid sequences are also treated as a specialized language. Therefore, the Transformer is also used to encode representations of proteins, thereby enhancing the performance of drug discovery [62,63].

Recently, variants of the Transformer have been gradually applied to various fields (see Section S7). In particular, the vision Transformer (ViT) has become a core architecture in the computer vision field. As shown in Fig. 4b, ViT retains the core principles of the original Transformer. In ViT, input images are divided into fixed-size patches as tokens, which are then linearly embedded into a sequence of vectors. Similar to ViT, the Transformer has also been extended to graph-structure data [64]. In particular, a graph Transformer, named Graphormer [65], has achieved competitive performance compared with GNNs. To leverage the topology of graphs, Graphormer incorporates *centrality encoding, spatial encoding* and *edge encoding* into Transformer, as shown in Fig. 4c. In light of Graphormer, the graph Transformer has emerged as a promising paradigm for graph data. Its key advantage lies in its ability to capture the relevance among nodes and enhance the topology in graphs, including node centrality, spatial distance and edge features. Vision and graph Transformers have been widely applied to drug discovery. For example, to capture 2D and 3D structures, some works, such as MAT [66], Transformer-M [67], MOLEBLEND [68] and Interformer [69], employed graph Transformers for drug discovery. TREE [64] proposed a Transformer-powered graph representation learning for cancer gene prediction, facilitating target identification in anti-caner drug discovery. In addition, in ISMol [70], 2D molecular graphs are treated as chemical-structure images and fed into a visual Transformer for property prediction.

## MULTIMODAL PRE-TRAINING TASKS

Pre-training task are among the most core components in self-supervised learning. Unimodal pre-training tasks (see Section S8), aim to guide models to learn the inherent representations and knowledge within the input data. Unlike unimodal tasks, multimodal pre-training objectives must account for alignment and fusion among different modalities. We summarize the difference between unimodal and multimodal pre-training tasks in Section S9. Here, we summarize four types of multimodal pre-training tasks in the field of drug discovery: contrastive learning [71,72], multimodal matching prediction [39,73], masked prediction [28,31] and autoregressive prediction [21,74]. The different pre-training tasks extract different features or attributes from the data, as shown in Table 2.

**Table 2.** Analysis and comparison of different multimodal self-supervised tasks.

Pre-training task	Optimization objective	Advantages and disadvantages
Multimodal contrastive learning	Modality alignment	$\bullet$ Capture the cross-modal consistency
		$\bullet$ Rely on the number of negative samples
		$\bullet$ Ignore the fine-grained alignment among modalities and specificity of each modality
Multimodal matching prediction	Consistency prediction	$\bullet$ Capture the cross-modal consistency and association
		$\bullet$ Rely on the quality of negative samples
		$\bullet$ Lose the specificity of each modality
Multimodal masked prediction	Context reconstruction	$\bullet$ Promote a deeper understanding of the fine-grained interaction among modalities
		$\bullet$ Ignore the cross-modal consistency
Multimodal autoregressive prediction	Condition generation	$\bullet$ Enhance the generation ability of models
		$\bullet$ Damage the representation ability of models
		$\bullet$ Ignore the cross-modal consistency

For a given entity, multimodal contrastive learning and matching prediction focus on capturing the consistency between various modalities, thus losing the specificity of each modality (see Sections S10– S11). The underlying principle of contrastive learning is to maximize agreement between positive samples and minimize agreement between negative samples [75,76]. Similarly, multi-modal matching prediction aims to learn high-quality representations by predicting whether a pair of samples from two modalities are matched (positive pair) or not matched (negative pair) [39]. Generally, in matching prediction and contrastive learning, instances of the same objects in different modalities are treated as positive pairs, and samples of different objects are treated as negative pairs. The key difference between matching prediction and contrastive learning is that the former maximizes the mutual information of positive pairs and minimize that of negative pairs, whereas the latter categorizes all samples as either positive or negative. Their performance is closely related to the number and quality of negative samples, respectively. For matching prediction, these positive and negative pairs are often easy to identify, which can limit its performance. In contrast, contrastive learning can avoid model collapse by using large-scale batches to generate sufficient negative samples. However, contrastive learning tasks fail to capture interaction or correspondences between modalities, whereas matching prediction tasks capture the cross-modal interactions by applying attention mechanisms or multilayer perceptrons after modality-specific encoders. Therefore, some studies, such as ISMol [70] and MolCA [77], have combined contrastive learning and matching prediction to achieve the complementary effect in drug discovery.

Masked prediction, initially developed for natural language processing by BERT [28], has emerged as a leading pre-training paradigm. The unimodal masked prediction task can improve a model’s ability to understand the internal features of input data. Recently, masked prediction tasks have been applied to multimodal molecular representation learning, where models predict masked information based on prompts from other modalities [70]. In cross-modal masked prediction, multiple types of input data are converted into unified sequences and then fed into an encoder [36,37]. Multimodal masked prediction ensures that models can understand the complex relationships and interactions between different modalities, further promoting the alignment of multimodal data. For single modality space, masked prediction focuses on the internal information within input data. However, multimodal masked prediction enables a deeper understanding of the fine-grained interaction among modalities, enhancing the representation ability.

Similar to masked prediction, autoregressive pre-training tasks [21,74] were initially proposed in natural language models and have been applied to SMILES sequence-based drug discovery (Section S13). In multimodal autoregressive prediction, data from different modalities are integrated into a unified sequence. Subsequently, autoregressive pre-training tasks predict the next tokens based on the preceding tokens in the unified sequences, preventing positions from attending to subsequent positions. Therefore, autoregressive pre-training tasks enhance the generative ability of models, but can impair their representation and understanding capabilities. Multiple studies have subsequently developed unified autoregressive models of text and molecules using wrapped sequences [40,78]. Similar to masked prediction, cross-modal autoregressive tasks ignore the consistency of each modality.

In the field of drug discovery, contrastive learning, matching prediction and masked prediction are more widely adopted than autoregressive tasks. This is mainly attributable to two reasons. First, autoregressive tasks utilize only unidirectional information, making them unable to capture the complete characteristics of molecules. Second, autoregressive tasks can enhance the generative ability of models. However, there are fewer generative tasks in the field of drug discovery. To further improve the performance of pre-training models, there have been increasing attempts to explore combinations of multiple pre-training tasks, leveraging their complementary strengths. This can be treated as a multi-task learning problem, in which a hybrid objective is often formulated as the weighted sum of multiple pre-training tasks. These pre-training tasks may be beneficial to different downstream tasks. Therefore, multi-task pre-training methods can lead to more flexible and general-purpose representations. However, combining multiple pre-training tasks increases hyperparameter complexity due to differing convergence rates and sample scales of the various tasks.

## SELF-SUPERVISED TRAINING STRATEGIES

**Figure 5.**
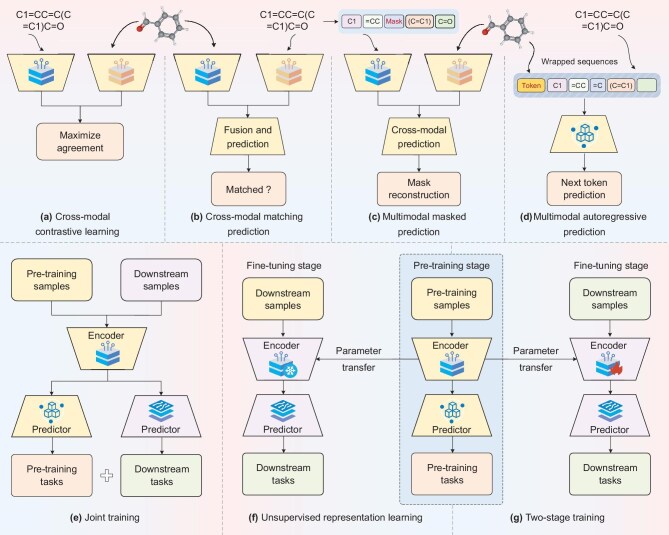
Multimodal self-supervised tasks (a–d) and training strategies (e–g).

The training strategies serve as a crucial bridge between the pre-training and fine-tuning stages. In Table 3, we summarize the key mechanisms of different training strategies. According to the relationships between pre-training and downstream tasks shown in Fig. 5(e–f), training strategies can be divided into three categories: joint training, unsupervised representation learning and two-stage training (pre-training and fine-tuning) [24,76].

**Table 3.** Analysis and comparison of different training strategies.

Pre-training strategy	Key role of pre-training model	Advantages and disadvantages
Joint training	Regularization or auxiliary term	$\bullet$ Improve the adaptability of pre-training models for current downstream tasks
		$\bullet$ Mitigate catastrophic forgetting problems
		$\bullet$ Reduce the generality and transferability of pre-training models
Unsupervised representation learning	Feature extraction	$\bullet$ Ensure the commonality and generality of pre-training algorithms
		$\bullet$ Mitigate catastrophic forgetting problems
		$\bullet$ Unable to meet the specific requirements of downstream tasks
Pre-training and fine-tuning	Parameter initialization of encoders	$\bullet$ Improve the adaptability of pre-training models for different downstream tasks
		$\bullet$ Ensure the commonality and generality of pre-training algorithms
		$\bullet$ Suffer from catastrophic forgetting and over-fitting problems

*Joint training.* In this scheme, the encoder is trained by combining self-supervised tasks and downstream tasks (see Section S14) [79,80]. Generally, the pre-training tasks serve as a regularization or auxiliary term for downstream tasks [24]. Intuitively, how to reasonably balance the loss values of pre-training and downstream tasks is a key factor in joint training. Joint training can enhance the adaptability of pre-training models to current downstream tasks and mitigate catastrophic forgetting. However, it reduces the generality and transferability of pre-training models.

*Unsupervised representation learning.* In this strategy, the encoder and self-supervised predictor are trained using pre-training tasks. The encoder is then frozen and added to the front of a new predictor for downstream tasks [38,81,82]. Therefore, the pre-training phase can be regarded as the feature-extraction process. In unsupervised representation learning, all downstream tasks share a pre-trained model, reducing computation while increasing flexibility and generality (see Section S15). Importantly, training only on prediction heads helps prevent overfitting when downstream tasks have small-scale training samples. However, freezing all pre-trained parameters prevents the model from capturing downstream task-specific features, although it mitigates catastrophic forgetting.

*Two-stage training.* Similar to unsupervised representation learning, encoders are trained by self-supervised tasks, thereby obtaining an encoder with high-quality parameters (see Section S16). Subsequently, the pre-trained encoder serves as the initial model in the fine-tuning stage and is fine-tuned together with the prediction head for downstream tasks. In two-stage training, the pre-training process can be considered a parameter-initialization step for the encoders. The pre-trained models are optimized to meet the requirements of downstream tasks, resulting in higher adaptability for different downstream tasks. However, as the number of pre-training model parameters grows, full parameter fine-tuning methods incurs a tremendous computational cost. Therefore, parameter-efficient fine-tuning techniques, including *additive, selective and reparameterized fine-tuning* methods, are applied in drug discovery [83]. These parameter-efficient fine-tuning algorithms aim to reduce the number of fine-tuning parameters while achieving performance comparable to full fine-tuning methods.

In the field of drug discovery, two-stage training strategies have become a general technique, whereas reparameterized fine-tuning remains an emerging technique. A common disadvantage of three self-supervised training strategies is the potential for negative transfer when the goals of pre-training and downstream tasks are weakly correlated or in conflict [84]. To address this issue, several studies have explored prompt-learning-based fine-tuning strategies to reduce the gap between pre-training and downstream tasks [34,85].

## DRUG DISCOVERY IN THE CONTEXT OF MULTIMODAL PRE-TRAINING

**Figure 6.**
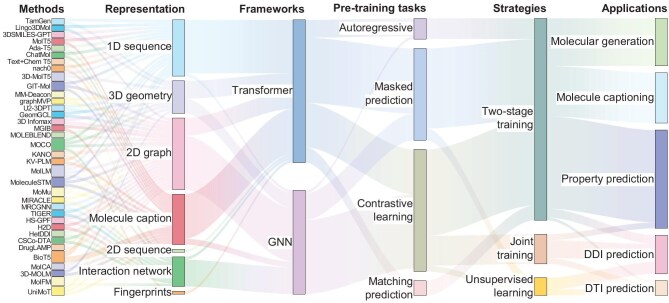
The application of multimodal pre-training in drug discovery. Note that this paper fails to summarize the development of pre-clinical studies, clinical trials and regulatory approval, as these mainly focus on cellular or patient responses.

This work focuses on summarizing multimodal pre-training models for molecular representation in drug discovery. Accordingly, we summarized the tasks related to lead discovery and optimization, including molecular generation, molecular property prediction, drug-drug interaction prediction, drug-target interaction prediction and molecule captioning. As shown in Fig. 6, an interesting observation is that the combination of 2D/3D molecular structure-based graph neural networks and SMILES-based Transformers constitutes a relatively popular framework in drug discovery. Masked prediction and contrast learning have emerged as the leading paradigms for molecular property prediction, DDI prediction and DTI prediction. Autoregressive prediction tasks are mainly applied to molecule generation and molecule captioning. With the successful application of large pre-trained models in natural language processing and computer vision, biomedical text-based multimodal pre-training models are mainly applied to molecule generation, molecular property prediction and molecule captioning. Simultaneously, molecule captioning has attracted significant attention from experts in the field of computer science. Most studies suggest that integrating multiple types of multimodal pre-training tasks is important for drug discovery. In addition, parameter-efficient fine-tuning-based drug discovery remains an emerging field. Here, we introduce the application of multimodal pre-training in drug discovery. further details can be found in Section S17.

### Molecular generation

Molecular generation aims to design new drugs with specific properties that include high affinity, safety and activity, low toxicity and structural novelty. In recent years, molecular generation has benefited from advances in multimodal pre-training. In drug discovery, 1D sequences are often treated as a chemical language system. It is therefore natural to develop GPT-like chemical language models for molecule generation, in which the Transformer decoder is pre-trained by autoregressively predicting the next SMILES tokens and fine-tuned using multimodal adapter mechanisms for target-aware molecular generation [63]. Along this line, atomic 3D coordinates are also treated as linguistic expressions and combined with SMILES to develop GPT-like autoregressive models [86]. In the fine-tuning stage, the pre-trained models often synergize with reinforcement learning to optimize the biophysical and chemical properties of the generated molecules. To further capture high-level structural information, Lingo3DMol [87] proposed an autoregressive pre-training approach that reconstructs the perturbed molecule back to its original state in both 2D and 3D representations. In the fine-tuning stage, the three encoder layers were fixed. The 2D decoder generates sequence fragments and local coordinates, while the other decoders generate 3D coordinates.

In addition, with the success of large pre-trained models in natural language processing, biomedical text-based molecular generation has also achieved significant progress. Based on a corpus consisting of SMILES sequences and textual descriptions, MolT5 [35] employed the recovery of masked spans—an extension of masked language models—to pre-train Transformer-based T5 [88]. Similarly, an increasing number of studies have begun to integrate textual descriptions and SMILES to pre-train Transformers [89–92]. These pre-training models are fine-tuned for molecule captioning and generation. To enable the seamless combination of molecular sequence and 3D structures, the fine-grained 3D structure-aware fingerprint is mapped into a specialized 3D token. Next, these specialized 3D tokens, molecular sequences and textual descriptions are fed into a T5-based unified architecture, pre-trained using masked-span recovery and cross-modal translation tasks. To fuse multimodal data, another studies have used multiple networks to encode SMILES, 2D molecule graphs, molecular images and captions, respectively. The multimodal neural networks are pre-trained using cross-modal contrastive learning and matching prediction, and prompt-tuned for molecule captioning and generation [93].

### Molecular property prediction

Molecular property prediction, a fundamental task in drug discovery, aims to infer the molecular attributes based on structure features. Multimodal pre-training techniques have been widely applied to molecular property prediction. There are two similar points among these methods. Firstly, most studies leveraged dual encoders that include SMILES-sequence-driven Transformer [60] and a 2D/3D-graph-structure-driven graph neural network [32,94]. Secondly, cross-modal contrastive learning is treated as a key pre-training task. In the fine-tuned stage, the task-specific prediction layer was attached to the pre-trained model for molecular property prediction. Inherently, contrastive-learning-based methods focus mainly on the consistency between different modalities, while ignoring the internal characteristics within each modality. To address this problem, more and more studies integrated multiple pre-training tasks, including contrastive learning, masked prediction and match prediction to improve the performance of property prediction [61,70,95]. In addition, the combination of more modalities, novel neural networks and domain knowledge is becoming an upward-trending research topic in molecular property prediction [33,34,68]. On the other hand, molecular-structure- and text-based pre-training models are applied to molecular property prediction. There are two types of popular paradigms. One is a deep-learning system that bridges molecular structures and biomedical texts [37]. To be specific, the molecular entities in biomedical texts are replaced with the segmented SMILES. Masked language models based on the mixed sequences encourage Transformers to capture the meta-knowledge among different semantic units, and are then fine-tuned for drug discovery, including molecular property prediction, named entity recognition and relation extraction. Other approaches often adopt multi-channel frameworks that integrate biomedical texts with SMILES and 2D and 3D molecular representations, and are pre-trained by contrastive learning [96–98]. Finally, the pre-trained models are fine-tuned for molecular property prediction.

### Drug-drug interaction prediction

Drugs may interact with each other when using drug combinations to treat diseases. Drug–drug interaction (DDI) increases the risk of adverse drug reactions. Therefore, it is significant to explore multimodal self-supervised learning-based DDI predictions. Similar to molecular property prediction, most multimodal methods employ contrastive learning frameworks for DDI predictions. However, most of these studies focus on using contrastive learning to align molecular structure and molecular interaction networks [38,79]. Simultaneously, advanced heterogeneous graph neural networks have been developed to capture multiple relationships within molecular interaction networks, improving the performance of DDI prediction [80,99]. Furthermore, a few works have synthesized 2D graphs and semantic information in biomedical interaction networks via attribute-masking and link-prediction tasks [100].

### Drug-target interaction prediction

Drug-target interaction (DTI) prediction aims to determine whether a given drug and target can interact with each other. Generally, DTI prediction is also treated as a drug-repositioning strategy that aims to screen potential drugs from existing ones for given targets [101]. Multimodal-based molecular pre-training is a potential technique in the field of DTI prediction. Based on SMILES and 2D molecular graphs, a growing body of work has employed masked prediction and contrastive learning to train Transformers and graph convolutional networks for drug–target interaction prediction [102]. Several works have learned features from molecular graphs and interaction networks via cross-modal graph-contrastive-learning approaches for drug–target affinity prediction, which is a similar task to DTI prediction [103]. On the other hand, BioT5 [36] integrates molecular SELFIES [104], protein sequences, general text wrapped text [40], in which molecule names were replaced with their corresponding SELFIES and gene names were appended with related protein sequences. To reduce the gap between pre-training and downstream tasks, BioT5 adopted prompt-based fine-tuning for drug-target interaction prediction and molecular property prediction.

### Molecule captioning

Given a molecular structure, molecule captioning aims to provide comprehensive text descriptions, enhancing the understanding of key characteristics that include the molecule name, chemical formula, basic structure properties and functions [105]. In comparison with other tasks in drug discovery, molecule captioning is still an emerging field. With the successful application of pre-training large models in natural language and computer vision, there is increasing interest in deep-learning-based molecule captioning. We find that molecule captioning and text-based molecule generation are often treated as bi-directional translation between molecules and language. The molecule-captioning task is similar to image-caption generation [106]. Most methods of molecule captioning refer to language-image pre-training models. A wide range of approaches employed multiple self-supervised tasks to train encoders that integrated GNNs and text-based pre-training models to learn representation from 2D molecular graphs and textual descriptions [107,108]. These multimodal pre-trained models are fully or parameter-efficiently fine-tuned for molecule captioning, IUPAC-name prediction and molecule-text retrieval. Following the line of BLIP-2 [106], recent works have utilized the Q-Former to bridge the gap between molecules and texts [109,110]. The Q-Former enables large language models to interpret and analyze molecules. These studies jointly pre-train the Q-Former together with the frozen encoder via multi-objective training.

## CONCLUSION AND OUTLOOK

In this paper, we present a comprehensiveness review of multimodal pre-training models for drug discovery, covering the spectrum from databases and molecular representations, through pre-training frameworks, tasks and strategies, to diverse applications. This review aims to discuss development of multimodal pre-training techniques in drug discovery and summarize novel perspectives. In the first place, we summarize the differences and relevance between various molecule representations. Then, we discuss different neural networks, and find that different modal representations are suitable for different network frameworks. In particular, molecular-sequence-based Transformers and 2D/3D-molecular-structure-based graph neural networks have emerged as the leading frameworks for drug discovery. Subsequently, we explore self-supervised tasks and pre-training strategies, highlighting the goals of different tasks and the trends of multi-modal and multi-task collaboration. Finally, we find that biomedical text provides a bridge for the integration of drug discovery with foundation models. Despite the significant progress, there are still challenges. In this section, we discuss the existing challenges and the future opportunities.

*Designing unified and flexible multi-modal frameworks.* In drug discovery, most multimodal approaches utilize Transformers and graph neural networks to encode molecular sequences and 2D/3D molecule structures, and then align different modalities via contrast learning or match prediction. Unfortunately, these coarse-grained alignment strategies miss the deep comprehension of items within different modalities [68]. On the other hand, graph neural networks often suffer from the over-smoothing phenomenon. In light of this, it is necessary to design unified pre-training frameworks that can fully encode features of each modality and ensure fine-grained alignment of various modalities. We may be able to refer to previous works, such as 3D-MolT5 [111], VLMO [112], SAPROT [113] and BEiT-3 [114], in which different modalities are converted into sequences and then fed into Transformers to learn representations. In addition, in practical scenarios, there is a high likelihood that some samples are missing certain modalities. Therefore, it is necessary to develop flexible frameworks to automatically handle cases with missing modalities.

*Discriminating consistency and complementarity among various modalities.* It is intuitive that there is not only complementarity but also consistency among multimodal representations of given molecules. Complementarity implies that each modality essentially has its specific or distinctive characteristics. In contrast, consistency suggests that there are shared attributes between different modality representations. Theoretically, multi-task pre-training approaches can capture cross-modal consistency and complementarity by integrating masked prediction, contrastive learning and matching prediction [33,70]. However, the major limitation of general multi-task frameworks is that the shared space may contain unnecessary specific features, and vice versa. In other words, general multi-task frameworks increase redundant information and computational complexity to a certain extent. Therefore, based on multi-task learning frameworks, determining how to discriminate and fuse consistency and complementarity among various modalities is crucial for improving the performance and efficiency of drug discovery.

*Incorporating more modalities and domain knowledge.* With the rapid development of life sciences, advanced techniques, such as molecular dynamics, single-cell sequencing techniques and high-throughput microscopies, generate more biomedical data with different modalities, including molecular videos [115], single-cell RNA sequencing data [116,117] and cell images [118], further promoting the application of multimodal pre-training models in the field of drug discovery. In addition, multimodal pre-training techniques can learn a variety of hidden information from these data. However, these deep-learning models find it difficult to automatically extract biochemical knowledge, which plays a key role in drug discovery. With the rapid development of life sciences and pharmacology, there is a wealth of biochemical knowledge, such as functional groups [119], coarse-grained model representations [120], motifs [121] and synthetic accessibility [122], that is closely related to molecular representations. The integration of pre-training models with such biochemical knowledge represents a significant opportunity to further advance drug discovery. Simultaneously, domain knowledge can enhance the interpretability of multimodal pre-training algorithms.

*Integrating general-purpose foundation models.* In the field of natural language processing, foundation models have made significant progress and have promoted the development of biomedical text mining. Being aware of this issue, a few studies, such as MolT5 [35] and BioT5 [36], incorporated textual descriptions and molecule SMILES to improve the performance of drug discovery. Unfortunately, these models only integrated 1D structure features, limiting the performance of drug discovery. On the other hand, compared to these specialized pre-trained models, general foundation models can learn the complex logic and context in long documents, resulting in a deeper understanding of the knowledge behind long texts. In addition, the corpus of general foundation models is more abundant and includes texts, images, audio and videos, providing interdisciplinary knowledge. Therefore, the fusion of general large language models with multimodal pre-training models opens new avenues for drug discovery and remains an open topic.

## Supplementary Material

nwaf495_Supplemental_File

## Data Availability

The key references investigated in this review can be accessed at https://github.com/AISciLab/MultiPM4Drug.git. In the online supplementary material, we summarize the molecular modalities, self-supervised tasks and downstream tasks of the cited methods.

## References

[1] Zhang P, Wang X, Cen X et al. A deep learning framework for in silico screening of anticancer drugs at the single-cell level. Natl Sci Rev 2025; 12: nwae451. 10.1093/nsr/nwae451 39872221 PMC11771446

[2] Zhang K, Yang X, Wang Y et al. Artificial intelligence in drug development. Nat Med 2025; 31: 45–59. 10.1038/s41591-024-03434-4 39833407

[3] Harrer S, Shah P, Antony B et al. Artificial intelligence for clinical trial design. Trends Pharmacol Sci 2019; 40: 577–91. 10.1016/j.tips.2019.05.005 31326235

[4] Nosengo N. New tricks for old drugs: faced with skyrocketing costs for developing new drugs, researchers are looking at ways to repurpose older ones–and even some that failed in initial trials. Nature 2016; 534: 314–7. 10.1038/534314a 27306171

[5] Liu T, Lu D, Zhang H et al. Applying high-performance computing in drug discovery and molecular simulation. Natl Sci Rev 2016; 3: 49–63. 10.1093/nsr/nww003 32288960 PMC7107815

[6] Tang X, Lei X, Liu L. A multi-modal drug target affinity prediction based on graph features and pre-trained sequence embeddings. Interdiscip Sci Comput Life Sci 2025; 17: 822–43. 10.1007/s12539-025-00713-7 40455402

[7] Jumper J, Evans R, Pritzel A et al. Highly accurate protein structure prediction with alphafold. Nature 2021; 596: 583–9. 10.1038/s41586-021-03819-2 34265844 PMC8371605

[8] Zhou X, Hu J, Zhang C et al. Assembling multidomain protein structures through analogous global structural alignments. Proc Natl Acad Sci USA 2019; 116: 15930–8. 10.1073/pnas.1905068116 31341084 PMC6689945

[9] Zhao K, Xia Y, Zhang F et al. Protein structure and folding pathway prediction based on remote homologs recognition using pathreader. Commun Biol 2023; 6: 243. 10.1038/s42003-023-04605-8 36871126 PMC9985440

[10] Zhao K, Zhao P, Wang S et al. FoldPAthreader: predicting protein folding pathway using a novel folding force field model derived from known protein universe. Genome Biol 2024; 25: 152. 10.1186/s13059-024-03291-x 38862984 PMC11167914

[11] He K, Zhang X, Ren S et al. Spatial pyramid pooling in deep convolutional networks for visual recognition. IEEE Trans Pattern Anal Mach Intell 2015; 37: 1904–16. 10.1109/TPAMI.2015.2389824 26353135

[12] Krizhevsky A, Sutskever I, Hinton GE. ImageNet classification with deep convolutional neural networks. Commun ACM 2017; 60: 84–90. 10.1145/3065386

[13] Chung J, Gulcehre C, Cho K et al. Empirical evaluation of gated recurrent neural networks on sequence modeling [preprint]. arXiv:1412.355.

[14] Hochreiter S, Schmidhuber J. Long short-term memory. Neural Comput 1997; 9: 1735–80. 10.1162/neco.1997.9.8.1735 9377276

[15] Kipf TN, Welling M. Semi-supervised classification with graph convolutional networks. 5th International Conference on Learning Representations, Toulon, France, 24–26 April 2017.

[16] Hamilton W, Ying Z, Leskovec J. Inductive representation learning on large graphs. In: Proceedings of the 31st International Conference on Neural Information Processing Systems. Red Hook, NY: Curran Associates, 2017, 1024–35.

[17] Veličković P, Cucurull G, Casanova A et al. Graph attention networks. 6th International Conference on Learning Representations, Vancouver, 30 April–3 May 2018.

[18] Wang N, Zhao S, Li Z et al. WDGBANDTI: a deep graph convolutional network-based bilinear attention network for drug-target interaction prediction with domain adaptation. Interdiscip Sci Comput Life Sci 2025; 17: 998–1017. 10.1007/s12539-025-00714-6 40410523

[19] Vaswani A, Shazeer N, Parmar N et al. Attention is all you need. In: Proceedings of the 31st International Conference on Neural Information Processing Systems. Red Hook, NY: Curran Associates, 2017, 6000–10.

[20] Wang X, Cheng Y, Yang Y et al. Multitask joint strategies of self-supervised representation learning on biomedical networks for drug discovery. Nat Mach Intell 2023; 5: 445–56. 10.1038/s42256-023-00640-6

[21] Brown T, Mann B, Ryder N et al. Language models are few-shot learners. In: Proceedings of the 34th International Conference on Neural Information Processing Systems. Red Hook, NY: Curran Associates, 2020, 1877–901.

[22] Chowdhery A, Narang S, Devlin J et al. PaLM: scaling language modeling with pathways. J Mach Learn Res 2023; 24: 11324–436.

[23] Guo F, Guan R, Li Y et al. Foundation models in bioinformatics. Natl Sci Rev 2025; 12: nwaf028. 10.1093/nsr/nwaf028 40078374 PMC11900445

[24] Liu Y, Jin M, Pan S et al. Graph self-supervised learning: A survey. IEEE Trans Knowl Data Eng 2022; 35: 5879–900.

[25] Su X, Wang Y, Gao S et al. KGARevion: an AI agent for knowledge-intensive biomedical QA. 13th International Conference on Learning Representations, Singapore: OpenReview, 24–28 April 2025.

[26] Chen Z, Tian S, Pei J et al. UniCure: a foundation model for predicting personalized cancer therapy response [preprint]. bioRxiv: 2025.06.14.658531.

[27] Xia J, Zhu Y, Du Y et al. A systematic survey of chemical pre-trained models. In: Proceedings of the Thirty-Second International Joint Conference on Artificial Intelligence. International Joint Conferences on Artificial Intelligence, 2023, 6787–95.

[28] Devlin J, Chang MW, Lee K et al. BERT: pre-training of deep bidirectional transformers for language understanding. In: Proceedings of the 2019 Conference of the North American Chapter of the Association for Computational Linguistics: Human Language Technologies. Stroudsburg, PA: Association for Computational Linguistics, 2019, 4171–86.

[29] Weininger D. SMILES, a chemical language and information system. 1. Introduction to methodology and encoding rules. J Chem Inf Comput Sci 1988; 28: 31–6.

[30] Ross J, Belgodere B, Chenthamarakshan V et al. Large-scale chemical language representations capture molecular structure and properties. Nat Mach Intell 2022; 4: 1256–64.

[31] Chithrananda S, Grand G, Ramsundar B. ChemBERTa: large-scale self-supervised pretraining for molecular property prediction [preprint]. arXiv: 2010.09885.

[32] Liu S, Wang H, Liu W et al. Pre-training molecular graph representation with 3D geometry. 10th International Conference on Learning Representations, Virtual, OpenReview, 25–29 April 2022.

[33] Zhu Y, Chen D, Du Y et al. Molecular contrastive pretraining with collaborative featurizations. J Chem Inf Model 2024; 64: 1112–22. 10.1021/acs.jcim.3c01468 38315002

[34] Fang Y, Zhang Q, Zhang N et al. Knowledge graph-enhanced molecular contrastive learning with functional prompt. Nat Mach Intell 2023; 5: 542–53. 10.1038/s42256-023-00654-0

[35] Edwards C, Lai T, Ros K et al. Translation between molecules and natural language. In: Proceedings of the 2022 Conference on Empirical Methods in Natural Language Processing. Stroudsburg, PA: Association for Computational Linguistics, 2022, 375–413. 10.18653/v1/2022.emnlp-main.26

[36] Pei Q, Zhang W, Zhu J et al. BioT5: enriching cross-modal integration in biology with chemical knowledge and natural language associations. In: Proceedings of the 2023 Conference on Empirical Methods in Natural Language Processing. Stroudsburg, PA: Association for Computational Linguistics, 2023, 1102–23. 10.18653/v1/2023.emnlp-main.70

[37] Zeng Z, Yao Y, Liu Z et al. A deep-learning system bridging molecule structure and biomedical text with comprehension comparable to human professionals. Nat Commun 2022; 13: 862. 10.1038/s41467-022-28494-3 35165275 PMC8844428

[38] Wang Y, Min Y, Chen X et al. Multi-view graph contrastive representation learning for drug-drug interaction prediction. In: Proceedings of the Web Conference 2021. New York: Association for Computing Machinery, 2021, 2921–33.

[39] Zong Y, Mac Aodha O, Hospedales T. Self-supervised multimodal learning: a survey. IEEE Trans Pattern Anal Mach Intell 2024; 47: 5299–318. 10.1109/TPAMI.2024.3429301 39110564

[40] Liu Z, Zhang W, Xia Y et al. MolXPT: wrapping molecules with text for generative pre-training. In: Proceedings of the 61st Annual Meeting of the Association for Computational Linguistics. Stroudsburg, PA: Association for Computational Linguistics, 2023, 1606–16.

[41] Bento AP, Hersey A, Félix E et al. An open source chemical structure curation pipeline using RDKit. J Cheminf 2020; 12: 51. 10.1186/s13321-020-00456-1 PMC7458899 33431044

[42] O’Boyle NM, Banck M, James CA et al. Open babel: an open chemical toolbox. J Cheminf 2011; 3: 33. 10.1186/1758-2946-3-33 PMC3198950 21982300

[43] Ektefaie Y, Dasoulas G, Noori A et al. Multimodal learning with graphs. Nat Mach Intell 2023; 5: 340–50. 10.1038/s42256-023-00624-6 38076673 PMC10704992

[44] Sun H, Wang J, Wu H et al. A multimodal deep learning framework for predicting PPI-modulator interactions. J Chem Inf Model 2023; 63: 7363–72. 10.1021/acs.jcim.3c01527 38037990

[45] Hu R, Ge R, Deng G et al. MultiKD-DTA: enhancing drug-target affinity prediction through multiscale feature extraction. Interdiscip Sci Comput Life Sci 2025; 17: 555–65. 10.1007/s12539-025-00697-4 40019659

[46] LeCun Y, Bengio Y, Hinton G. Deep learning. Nature 2015; 521: 436–44. 10.1038/nature14539 26017442

[47] Rifaioglu AS, Nalbat E, Atalay V et al. Deepscreen: high performance drug–target interaction prediction with convolutional neural networks using 2-D structural compound representations. Chem Sci 2020; 11: 2531–57. 10.1039/C9SC03414E 33209251 PMC7643205

[48] Fernandez M, Ban F, Woo G et al. Toxic colors: the use of deep learning for predicting toxicity of compounds merely from their graphic images. J Chem Inf Model 2018; 58: 1533–43. 10.1021/acs.jcim.8b00338 30063345

[49] Zeng X, Xiang H, Yu L et al. Accurate prediction of molecular properties and drug targets using a self-supervised image representation learning framework. Nat Mach Intell 2022; 4: 1004–16. 10.1038/s42256-022-00557-6

[50] Kiranyaz S, Avci O, Abdeljaber O et al. 1d convolutional neural networks and applications: a survey. Mech Syst Sig Process 2021; 151: 107398. 10.1016/j.ymssp.2020.107398

[51] Zhou J, Cui G, Hu S et al. Graph neural networks: a review of methods and applications. AI Open 2020; 1: 57–81. 10.1016/j.aiopen.2021.01.001

[52] Schütt KT, Sauceda HE, Kindermans PJ et al. Schnet–a deep learning architecture for molecules and materials. J Chem Phys 2018; 148. 10.1063/1.5019779 29960322

[53] Schütt KT, Arbabzadah F, Chmiela S et al. Quantum-chemical insights from deep tensor neural networks. Nat Commun 2017; 8: 13890. 10.1038/ncomms13890 28067221 PMC5228054

[54] Zhang C, Song D, Huang C et al. Heterogeneous graph neural network. In: Proceedings of the 25th ACM SIGKDD International Conference on Knowledge Discovery & Data Mining. New York: Association for Computing Machinery, 2019, 793–803.

[55] Wang X, Ji H, Shi C et al. Heterogeneous graph attention network. In: The World Wide Web Conference. New York: Association for Computing Machinery, 2019, 2022–32. 10.1145/3308558.3313562

[56] Rusch TK, Bronstein MM, Mishra S. A survey on oversmoothing in graph neural networks [preprint]. arXiv: 2303.10993.

[57] Wang X, Wen Y, Zhang Y et al. A hierarchical attention network integrating multi-scale relationship for drug response prediction. Inform Fusion 2024; 110: 102485. 10.1016/j.inffus.2024.102485

[58] He K, Zhang X, Ren S et al. Deep residual learning for image recognition. In: Proceedings of the IEEE Conference on Computer Vision and Pattern Recognition. Piscataway, NJ: IEEE Press, 2016, 770–8.

[59] Ba JL, Kiros JR, Hinton GE. Layer normalization [preprint]. arXiv: 1607.06450.

[60] Guo Z, Sharma PK, Martinez A et al. Multilingual molecular representation learning via contrastive pre-training. In: Proceedings of the 60th Annual Meeting of the Association for Computational Linguistics. Stroudsburg, PA: Association for Computational Linguistics, 2022, 3441–53.

[61] Zhu J, Xia Y, Wu L et al. Dual-view molecular pre-training. In: Proceedings of the 29th ACM SIGKDD Conference on Knowledge Discovery and Data Mining. New York: Association for Computing Machinery, 2023, 3615–27.

[62] Chen L, Fan Z, Chang J et al. Sequence-based drug design as a concept in computational drug design. Nat Commun 2023; 14: 4217. 10.1038/s41467-023-39856-w 37452028 PMC10349078

[63] Wu K, Xia Y, Deng P et al. TamGen: drug design with target-aware molecule generation through a chemical language model. Nat Commun 2024; 15: 9360. 10.1038/s41467-024-53632-4 39472567 PMC11522292

[64] Su X, Hu P, Li D et al. Interpretable identification of cancer genes across biological networks via transformer-powered graph representation learning. Nat Biomed Eng 2025; 9: 371–89. 10.1038/s41551-024-01312-5 39789329

[65] Ying C, Cai T, Luo S et al. Do transformers really perform badly for graph representation? In: 34th Annual Conference on Advances in Neural Information Processing Systems. Red Hook, NY: Curran Associates, 2021, 28877–88.

[66] Maziarka Ł, Danel T, Mucha S et al. Molecule attention transformer [preprint]. arXiv: 2002.08264.

[67] Luo S, Chen T, Xu Y et al. One transformer can understand both 2D & 3D molecular data. 11th International Conference on Learning Representations, Kigali, Rwanda: OpenReview, 1–5 May 2023.

[68] Yu Q, Zhang Y, Ni Y et al. Multimodal molecular pretraining via modality blending. 12th International Conference on Learning Representations, Vienna, Austria: OpenReview, 7–11 May 2024.

[69] Lai H, Wang L, Qian R et al. Interformer: an interaction-aware model for protein-ligand docking and affinity prediction. Nat Commun 2024; 15: 10223. 10.1038/s41467-024-54440-6 39587070 PMC11589619

[70] Zhang X, Xiang H, Yang X et al. Dual-view learning based on images and sequences for molecular property prediction. IEEE J Biomed Health Inform 2023; 28: 1564–74. 10.1109/JBHI.2023.3347794 38153823

[71] Radford A, Kim JW, Hallacy C et al. Learning transferable visual models from natural language supervision. In: Proceedings of the 38th International Conference on Machine Learning (Proc Mach Learn Res 139). JMLR, 2021, 8748–63.

[72] Pinheiro GA, Da Silva JL, Quiles MG. Smiclr: contrastive learning on multiple molecular representations for semisupervised and unsupervised representation learning. J Chem Inf Model 2022; 62: 3948–60. 10.1021/acs.jcim.2c00521 36044610

[73] Chen YC, Li L, Yu L et al. UNITER: universal Image-TExt Representation Learning In: Vedaldi A, Bischof H, Brox T, Frahm JM (eds.) Computer Vision–ECCV 2020. Cham: Springer, 2020, 104–20.

[74] Dai Z, Yang Z, Yang Y et al. Transformer-XL: attentive language models beyond a fixed-length context. In: Proceedings of the 57th Annual Meeting of the Association for Computational Linguistics. Stroudsburg, PA: Association for Computational Linguistics, 2019, 2978–88. 10.18653/v1/P19-1285

[75] Chen T, Kornblith S, Norouzi M et al. A simple framework for contrastive learning of visual representations. In: Proceedings of the 37th International Conference on Machine Learning (Proc Mach Learn Res 119). JMLR, 2020, 1597–607.

[76] Wu L, Lin H, Tan C et al. Self-supervised learning on graphs: contrastive, generative, or predictive. IEEE Trans Knowl Data Eng 2021; 35: 4216–35. 10.1109/TKDE.2021.3131584

[77] Liu Z, Li S, Luo Y et al. MolCA: molecular graph-language modeling with cross-modal projector and uni-modal adapter. In: Proceedings of the 2023 Conference on Empirical Methods in Natural Language Processing. Stroudsburg, PA: Association for Computational Linguistics, 2023, 15623–38. 10.18653/v1/2023.emnlp-main.966

[78] Bagal V, Aggarwal R, Vinod P et al. MolGPT: molecular generation using a transformer-decoder model. J Chem Inf Model 2021; 62: 2064–76. 10.1021/acs.jcim.1c00600 34694798

[79] Xiong Z, Liu S, Huang F et al. Multi-relational contrastive learning graph neural network for drug-drug interaction event prediction. In: Proceedings of the AAAI Conference on Artificial Intelligence. Washington, DC: Association for the Advancement of Artificial Intelligence, 2023, 5339–47. 10.1609/aaai.v37i4.25665

[80] Su X, Hu P, You ZH et al. Dual-channel learning framework for drug-drug interaction prediction via relation-aware heterogeneous graph transformer. In: Proceedings of the AAAI Conference on Artificial Intelligence. Washington, DC: Association for the Advancement of Artificial Intelligence, 2024, 249–56. 10.1609/aaai.v38i1.27777

[81] Wang X, Xin B, Tan W et al. DeepR2cov: deep representation learning on heterogeneous drug networks to discover anti-inflammatory agents for covid-19. Brief Bioinform 2021; 22: bbab226. 10.1093/bib/bbab226 34117734 PMC8344611

[82] Wang X, Yang Y, Li K et al. BioERP: biomedical heterogeneous network-based self-supervised representation learning approach for entity relationship predictions. Bioinformatics 2021; 37: 4793–800. 10.1093/bioinformatics/btab565 34329382

[83] Han Z, Gao C, Liu J et al. Parameter-efficient fine-tuning for large models: a comprehensive survey [preprint]. arXiv: 2403.14608.

[84] Hu W, Liu B, Gomes J et al. Strategies for pre-training graph neural networks. 8th International Conference on Learning Representations, Virtual, OpenReview, 26 April–1 May 2020.

[85] Ye Y, Zhou J, Li S et al. Hierarchical structure-aware graph prompting for drug-drug interaction prediction. In: Machine Learning and Knowledge Discovery in Databases. Cham: Springer, 2024, 36–54.

[86] Wang J, Luo H, Qin R et al. 3DSMILES-GPT: 3D molecular pocket-based generation with token-only large language model. Chem Sci 2025; 16: 637–48. 10.1039/D4SC06864E 39664804 PMC11629531

[87] Feng W, Wang L, Lin Z et al. Generation of 3D molecules in pockets via a language model. Nat Mach Intell 2024; 6: 62–73. 10.1038/s42256-023-00775-6

[88] Raffel C, Shazeer N, Roberts A et al. Exploring the limits of transfer learning with a unified text-to-text transformer. J Mach Learn Res 2020; 21: 5485–551.

[89] Chen Y, Xi N, Du Y et al. From artificially real to real: Leveraging pseudo data from large language models for low-resource molecule discovery. In: Proceedings of the AAAI Conference on Artificial Intelligence. Washington, DC: Association for the Advancement of Artificial Intelligence, 2024, 21958–66. 10.1609/aaai.v38i20.30198

[90] Zeng Z, Yin B, Wang S et al. ChatMol: interactive molecular discovery with natural language. Bioinformatics 2024; 40: btae534. 10.1093/bioinformatics/btae534 39222004 PMC11520398

[91] Christofidellis D, Giannone G, Born J et al. Unifying molecular and textual representations via multi-task language modelling. In: Proceedings of the 40th International Conference on Machine Learning (Proc Mach Learn Res 202). JMLR, 2023, 6140–57.

[92] Livne M, Miftahutdinov Z, Tutubalina E et al. nach0: multimodal natural and chemical languages foundation model. Chem Sci 2024; 15: 8380–9. 10.1039/D4SC00966E 38846388 PMC11151847

[93] Liu P, Ren Y, Tao J et al. GIT-Mol: a multi-modal large language model for molecular science with graph, image, and text. Comput Biol Med 2024; 171: 108073. 10.1016/j.compbiomed.2024.108073 38359660

[94] Stärk H, Beaini D, Corso G et al. 3d infomax improves gnns for molecular property prediction. In: Proceedings of the 39th International Conference on Machine Learning (Proc Mach Learn Res 162). JMLR, 2022, 20479–502.

[95] Zang X, Zhang J, Tang B. Self-supervised pre-training via multi-view graph information bottleneck for molecular property prediction. IEEE J Biomed Health Inform 2024; 28: 7659–69. 10.1109/JBHI.2024.3422488 38959149

[96] Tang X, Tran A, Tan J et al. MolLM: a unified language model for integrating biomedical text with 2D and 3D molecular representations. Bioinformatics 2024; 40: i357–68. 10.1093/bioinformatics/btae260 38940177 PMC11256921

[97] Liu S, Nie W, Wang C et al. Multi-modal molecule structure–text model for text-based retrieval and editing. Nat Mach Intell 2023; 5: 1447–57. 10.1038/s42256-023-00759-6

[98] Su B, Du D, Yang Z et al. A molecular multimodal foundation model associating molecule graphs with natural language [preprint]. arXiv: 2209.05481.

[99] Zhang R, Wang X, Wang S et al. H2D: hierarchical heterogeneous graph learning framework for drug-drug interaction prediction. In: Proceedings of the 33rd ACM International Conference on Information and Knowledge Management. New York: Association for Computing Machinery, 2024, 4283–7.

[100] Li Z, Tu X, Chen Y et al. HetDDI: a pre-trained heterogeneous graph neural network model for drug–drug interaction prediction. Brief Bioinform 2023; 24: bbad385. 10.1093/bib/bbad385 37903412

[101] Ashburn TT, Thor KB. Drug repositioning: identifying and developing new uses for existing drugs. Nat Rev Drug Discov 2004; 3: 673–83. 10.1038/nrd1468 15286734

[102] Luo Z, Wu W, Sun Q et al. Accurate and transferable drug–target interaction prediction with druglamp. Bioinformatics 2024; 40: btae693. 10.1093/bioinformatics/btae693 39570605 PMC11629708

[103] Wang J, Xiao Y, Shang X et al. Predicting drug–target binding affinity with cross-scale graph contrastive learning. Brief Bioinform 2024; 25: bbad516. 10.1093/bib/bbae516 PMC10788681 38221904

[104] Krenn M, Häse F, Nigam A et al. Self-referencing embedded strings (SELFIES): a 100% robust molecular string representation. Mach Learn Sci Technol 2020; 1: 045024. 10.1088/2632-2153/aba947

[105] Li J, Liu Y, Fan W et al. Empowering molecule discovery for molecule-caption translation with large language models: A chatgpt perspective. IEEE Trans Knowl Data Eng 2024; 36: 6071–83. 10.1109/TKDE.2024.3393356

[106] Li J, Li D, Savarese S et al. BLIP-2: Bootstrapping language-image pre-training with frozen image encoders and large language models. In: Proceedings of the 40th International Conference on Machine Learning (Proc Mach Learn Res 202). JMLR, 2023, 19730–42.

[107] Liu Z, Li S, Luo Y et al. MolCA: molecular graph-language modeling with cross-modal projector and uni-modal adapter. In: Proceedings of the 2023 Conference on Empirical Methods in Natural Language Processing. Stroudsburg, PA: Association for Computational Linguistics, 2023, 15623–38. 10.18653/v1/2023.emnlp-main.966

[108] Luo Y, Yang K, Hong M et al. MolFM: a multimodal molecular foundation model [preprint]. arXiv: 2307.09484.

[109] Li S, Liu Z, Luo Y et al. Towards 3D molecule-text interpretation in language models. 12th International Conference on Learning Representations, Vienna, Austria: OpenReview, 7–11 May 2024.

[110] Zhang J, Bian Y, Chen Y et al. UniMoT: unified molecule-text language model with discrete token representation [preprint]. arXiv: 2408.00863.

[111] Pei Q, Yan R, Gao K et al. 3D-MolT5: leveraging discrete structural information for molecule-text modeling [preprint]. arXiv: 2406.05797.

[112] Bao H, Wang W, Dong L et al. VLMo: unified vision-language pre-training with mixture-of-modality-experts. In: 35th Annual Conference on Advances in Neural Information Processing Systems. Red Hook, NY: Curran Associates, 2022, 32897–912.

[113] Su J, Han C, Zhou Y et al. SaProt: protein language modeling with structure-aware vocabulary. 12th International Conference on Learning Representations, Vienna, Austria: OpenReview, 7–11 May 2024.

[114] Wang W, Bao H, Dong L et al. Image as a foreign language: BEIT pretraining for vision and vision-language tasks. In: Proceedings of the IEEE/CVF Conference on Computer Vision and Pattern Recognition. Piscataway, NJ: IEEE Press, 2023, 19175–86.

[115] Xiang H, Zeng L, Hou L et al. A molecular video-derived foundation model for scientific drug discovery. Nat Commun 2024; 15: 9696. 10.1038/s41467-024-53742-z 39516468 PMC11549228

[116] Chen J, Wang X, Ma A et al. Deep transfer learning of cancer drug responses by integrating bulk and single-cell RNA-seq data. Nat Commun 2022; 13: 6494. 10.1038/s41467-022-34277-7 36310235 PMC9618578

[117] Liu X, Shi L, Zhao Z et al. VIBRANT: spectral profiling for single-cell drug responses. Nat Methods 2024; 21: 501–11. 10.1038/s41592-024-02185-x 38374266 PMC11214684

[118] Chandrasekaran SN, Ceulemans H, Boyd JD et al. Image-based profiling for drug discovery: due for a machine-learning upgrade? Nat Rev Drug Discov 2021; 20: 145–59. 10.1038/s41573-020-00117-w 33353986 PMC7754181

[119] Li B, Lin M, Chen T et al. FG-BERT: a generalized and self-supervised functional group-based molecular representation learning framework for properties prediction. Brief Bioinform 2023; 24: bbad398. 10.1093/bib/bbad398 37930026

[120] Yang W, Templeton C, Rosenberger D et al. Slicing and dicing: optimal coarse-grained representation to preserve molecular kinetics. ACS Cent Sci 2023; 9: 186–96. 10.1021/acscentsci.2c01200 36844497 PMC9951291

[121] Yu Z, Gao H. Molecular representation learning via heterogeneous motif graph neural networks. In: Proceedings of the 39th International Conference on Machine Learning (Proc Mach Learn Res 162). JMLR, 2022, 125581–94.

[122] Ertl P, Schuffenhauer A. Estimation of synthetic accessibility score of drug-like molecules based on molecular complexity and fragment contributions. J Cheminf 2009; 1: 8. 10.1186/1758-2946-1-8 PMC3225829 20298526

