# Supplementary material for: Multimodal pre-training models of molecular representation for drug discovery
Source: Natl Sci Rev. 2025 Nov 11;13(1):nwaf495. doi: 10.1093/nsr/nwaf495 (PMC12798728; doi:10.1093/nsr/nwaf495)
Supplement: nwaf495_Supplemental_File [file nwaf495_supplemental_file.pdf]

# Supplementary Materials for

# Multimodal Pre-training Models of Molecular Representation for Drug Discovery

**Xiaoqi Wang[1,2], Chuanshi Wang[1], Boya Ji[3], Junwen Wang[4], Mingyue Zheng[5], Lingyun Song[1,2], Shaoliang Peng[3*], and Xuequn Shang[1,2*]**

[1]School of Computer Science, Northwestern Polytechnical University, Xi'an, 710129, China.
[2]Key Laboratory of Big Data Storage and Management, Northwestern Polytechnical University, Ministry of Industry and Information Technology, Xi'an, 710129, China.
[3]College of Computer Science and Electronic Engineering, Hunan University, Changsha, 410082, China.
[4]Division of Applied Oral Sciences & Community Dental Care, Faculty of Dentistry, the University of Hong Kong, Hong Kong SAR, China.
[5]Drug Discovery and Design Center, State Key Laboratory of Drug Research, Shanghai Institute of Materia Medica, Chinese Academy of Sciences, Shanghai 201203, China

**The Supplementary Material file includes**


- S1. Representative databases and molecular representation
- S2. Deep neural networks
- S3. Convolutional neural networks
- S4. Recurrent neural networks
- S5. Graph neural networks
- S6. Transformer
- S7. Vision and graph Transformers
- S8. Unimodal pre-training tasks
- S9. Comparison unimodal and multimodal pre-training tasks
- S10. Cross-modal contrastive learning
- S11. Multimodal matching prediction
- S12. Multimodal masked prediction
- S13. Multimodal autoregressive prediction
- S14. Joint training
- S15. Unsupervised representation learning
- S16. Two-stage training
- S17. Application of multimodal pre-training models in drug discovery
- S18. Summary of all cited methods

# S1. Representative databases and molecular representation

## Databases

The growth of biomedical data provides vast opportunities for deep learning-based drug discovery. The scale and quality of biomedical data is the key factors for successful application of deep learning in the field of drug discovery. Therefore, a series of free and open-access databases with structured biomedical data, is developed to promote the progress of drug discovery. We summarize several popular databases in Table S1, and provide a brief description. For example, DrugBank[1] compiles the chemistry and pharmacology data, including chemical structure, drug-drug interactions, drug targets, and biological pathways. The DrugBank (v5.1.13) version released in 2025 collected 5,134 targets and 17,449 drugs, in which includes 2,812 Food and Drug Administration-approved small molecules. ChEMBL[2] integrates biochemical activity and genomic data. Based on a foundation of 92,121 publications and 293 deposited datasets, ChEMBL (v35) compiles 2,496,335 compounds, 16,003 targets, and a wealth of 21,123,501 biological activity data. In addition, based on these basic databases, heterogeneous biomedical networks[3–5] are constructed to explore the diverse relationships among multiple biomedical entities. CKG[3] constructed a biological network that contains approximately 20 million biological entities and 200 million relationships from 25 biomedical databases. Similarly, PrimeKG[5] derived a network with 10 types of biological entities and 30 types of relationships by fusing 20 high-quality resources. These biological networks contain heterogeneous biological entities and relationships, which can better simulate living systems and further promote drug discovery.

## Molecular Representation

In the real world, molecules are encoded as different modalities including molecular descriptors, one-dimensional (1D) molecular sequences, 2D molecular graphs, 3D molecular structures, molecular interaction networks, and textual captions. These different modalities reflect the molecular features of different perspectives.

**1D molecular sequences:** In 1D sequences, each molecule is transformed into strings based on the bonding rules of chemical structures, such as simplified molecular-input line-entry system (SMILES),[6] IUPAC International Chemical Identifier (InChI),[7] and SMILES Arbitrary Target Specification (SMARTS),[8] In particular, SMILES has become one of most popular molecule sequences, because of its versatility and interpretability. In SMILES sequences, each atom is represented by its corresponding ASCII code, and chemical bonds, stereochemistry, and branches that are denoted via specific symbols. However, the initial atoms are often randomly chosen in productive processes of SMILES. Therefore, for a given molecule, there are multiple SMILES strings that lay the foundation for data augmentation and can improve the robustness of deep learning. **2D molecular graphs:** Molecules can also be represented as 2D graphs in which atoms and bonds are treated as nodes and edges, respectively. 2D molecular graphs can reflect the topological relationships among atoms. Concurrently, nodes and edges are also encoded as vectors that represent the atom or bond attributes. Therefore, molecular graphs are often described via node feature matrices, edge feature matrices, and adjacency matrices. Node features primarily provide various atom information including atom types, aromaticity, and partial charge, while edge features provide bond information including bond types, bond directions, and rings.[9] **Fingerprints:** Similar to molecular graphs, molecular fingerprints also reflect the molecular topology in 2D space.[10] However, molecular fingerprints encode 2D structure of molecules into binary vectors, such as PubChem fingerprint,[11] MACCS fingerprint,[12] and ECFP fingerprint.[13] In molecular fingerprints, each element value (1 or 0) indicates the presence or absence of specific substructures in molecules. **3D molecular geometries:** For a given molecule, 3D geometry represents the arrangements of atoms in 3D space, providing the geometric position of each atom. Concurrently, it is also possible to directly leverage stereochemistry features. In other words, 3D geometries can capture the conformer information that is important to molecular properties.[14] In addition, the spherical coordinate system based on bond lengths, bond angles, and torsion angles can also be treated as the 3D geometric representation of molecules.[15] **Molecular descriptor:** It employs mathematical methods to quantitatively represent physical or chemical properties including molecular weight, atomic number, and atom type counts.[16] Molecular descriptors are encoded as continuous or discrete values. It is worth noting that there may be significant correlations between physicochemical properties. Therefore, feature selection or dimension reduction techniques are often used to extract key information from molecular descriptors. **Molecular interaction networks**: Molecular networks denote the complex relationships between drugs and biomedical entities, such as genes, proteins, and diseases, reflecting the biological processes in the human body. For example, drug-drug interactions occur frequently when concurrently taking two or multiple drugs, causing adverse drug reactions or diseasees.[17] Therefore, molecular interaction networks play a crucial role in understanding and discovering the mechanisms of action among drugs. **Molecule captions**: The captions of molecules typically refer to a brief text description that annotates key characteristics and properties. For a given molecule, its captions often introduce molecular names, chemical formulas, basic structures, etc.[18] In addition, the captions of molecules describe their properties and functions in biological or chemical processes. These molecule captions can help researchers and readers understand the molecular composition, structure, functions, or mechanisms of action.

**Table S1.** Representative databases for drug discovery

| Database | Website | Description |
|---|---|---|
| DrugBank[1] | http://www.drugbank.ca | DrugBank is comprehensive database containing information on drugs and targets. |
| PubChem[11] | https://pubchem.ncbi.nlm.nih.gov/ | PubChem provides detailed drug molecule information, including chemical structure and properties, biological activity, toxicity, and more. |
| ChEMBL[2] | https://www.ebi.ac.uk/chembl/ | ChEMBL contains biochemical activity and genomic data. |
| ZINC[19] | https://zinc15.docking.org/ | ZINC contains a great number of purchasable molecules with 3D structure, which have been assigned biologically relevant protonation states and annotated properties. |
| ChemDB[20] | https://cdb.ics.uci.edu/ | ChemDB is a chemical database that contains nearly 5 million commercially available molecules, along with their predicted or experimentally determined physicochemical properties. |
| SIDER[21] | http://sideeffects.embl.de/ | SIDER focuses on adverse reaction information of marketed medicines. |
| CTD[22] | https://ctdbase.org/ | Comparative Toxicogenomics Database (CTD) contains manually integrated information of chemical molecules, including interactions among molecules, genes diseases and phenotypes. |
| TTD[23] | https://db.idrblab.net/ttd/ | Therapeutic Target Database (TTD) collects information about drugs, targets (proteins and nucleic acids), diseases and pathways. |
| RepoDB[24] | http://unmtid-shinyapps.net/shiny/repodb/ | Repository of Promoting Data (RepoDB) collects drugs, diseases, and their relationships information. |
| BioGRID[25] | https://thebiogrid.org/ | BioGRID collects protein and genetic interaction information and chemical interaction networks. |
| PharmGKB[26] | https://www.pharmgkb.org/ | PharmGKB mainly focuses on information about drug-gene associations and genotype-phenotype relationships. |
| STITCH[27] | http://stitch.embl.de/ | STITCH provides drug-target interaction data. |
| DrugCentral[28] | https://drugcentral.org/ | DrugCentral provides information about pharmaceutical products, active ingredients chemical entities, pharmacologic action, indications, mechanism of action. |
| PRISM[29] | https://depmap.org/repurposing/ | PRISM contains drug response to cancer cell lines. |
| CCLE[30] | https://sites.broadinstitute.org/ccle | Cancer Cell Line Encyclopedia (CCLE) performs large-scale sequencing of human cancer cell lines, integrating information about drug response, DNA mutations, gene expression, and gene copy number. |
| GDSC[31] | https://www.cancerrxgene.org/ | Genomics of Drug Sensitivity in Cancer (GDSC) identifies drug sensitivity and molecular marker information of human cancer cell lines. |
| DrugCombDB[32] | http://drugcombdb.denglab.org/main | DrugCombDB contains a drug combinations data for cancer cell lines. |
| DrugComb[33] | https://drugcomb.org/ | DrugComb is a monotherapy response and drug combinations database. |
| GEO[34] | https://www.ncbi.nlm.nih.gov/geo/ | Gene Expression Omnibus (GEO) contains gene expression profile data for various biological samples around the world. |
| CKG[3] | https://ckg.readthedocs.io/en/latest/ | CKG (Clinical Knowledge Graph) comprises close to 20 million nodes and 220 million relationships |

## S2. Deep neural networks

Deep neural network (DNN) is a class of deep learning frameworks.[35] DNNs consist of an input layer that receives input data, an output layer that makes decisions or predictions, and one or multiple hidden layers, as shown in Figure S1(A). The principle of DNNs is as follows: Data is fed into the input layer, and then hidden layers process input data by the linear transformations and non-linear activation functions, e.g., ReLU, Tanh, Sigmoid, and Softmax. Finally, the output layer generates results based on the representation from hidden layers. Input data becomes more abstract as the deeper hidden layers. DNNs iteratively adjust thier own parameters by using backpropagation processes that aim to minimize the difference between predicted results and true values, enabling them to complete classification or regression tasks.[35] Deep neural networks can automatically learn complex and non-linear relationships behind data, and have improved the performance of drug discovery.[17,36,37]

## S3. Convolutional neural networks

Convolutional neural networks (CNNs) are primarily used for grid-like data.[38] As shown in Figure S1(B), CNNs consist of convolutional layers, pooling layers, and fully connected layers. Convolution layers extract features from input data, while pooling layers reduce feature dimensionality. The fully connected layers aim to complete prediction based on features from convolution or pooling layers. One line of CNN models, such as VGG,[39] AlexNet,[40] DenseNet,[41] and ResNet,[42] have been successfully applied to the field of computer vision and video. A distinctive advantage of CNNs is parameter sharing in each convolution layer, reducing the total number of parameters and speeding up computation. Therefore, numerous studies proposed 1D CNNs for sequential data by extending the principles of traditional convolutional networks.[43] In 1D CNNs, convolution operations are performed over a single spatial dimension to analyze sequential data. 1D CNNs have been used to encode molecular SMILES or fingerprints for drug discovery.[44,45] On the other hand, several studies hold that 2D molecular graphs can be treated as images, and then fed into 2D CNNs for drug discovery.[46–48]

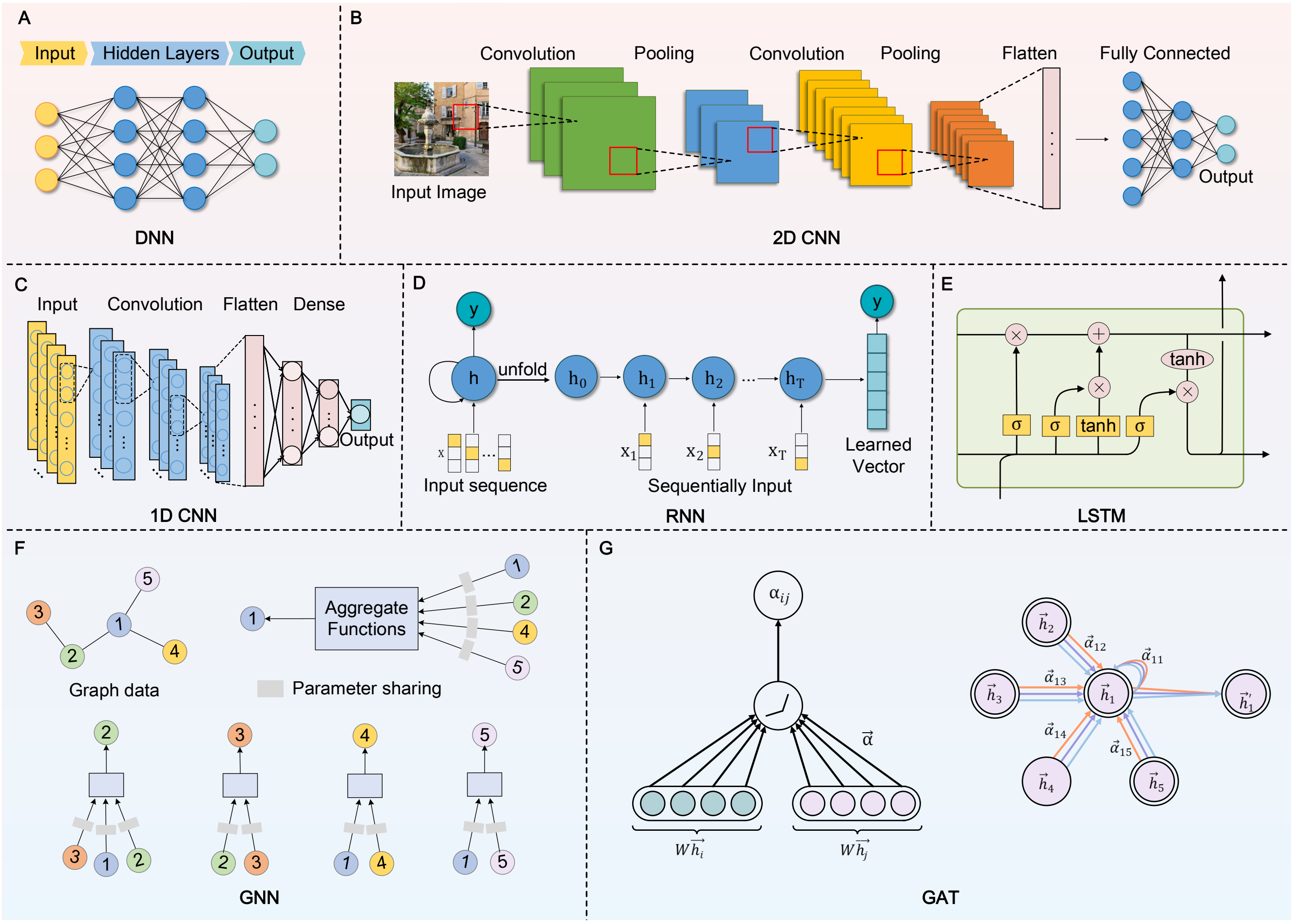


**Figure S1.** The classical frameworks of deep learning models

## S4. Recurrent neural networks

Recurrent neural networks (RNNs) are specifically designed for sequential or time-series data. RNNs incorporate directed cycles in hidden layers, enabling the output of one time step as the input for the next, as shown in Figure S1(D). This unique structure gives RNNs a form of "memory" about previous information.[35] Unlike DNNs and CNNs, where inputs and outputs are treated as independent, RNNs rely on the prior elements in sequence data for generating current outputs. However, RNNs suffer from the challenges vanishing or exploding gradients when the length of sequences becomes long. To address this problem, Long Short-Term Memory (LSTM) network[49] integrates special units called memory cells to maintain information for long periods. Each memory cell includes gates that regulate the flow of information. LSTM significantly improves performance on tasks of long sequences. In addition, advanced models like Gated Recurrent Units (GRU)[50] and bidirectional recurrent neural networks (BRNNs)[51] are designed to enable better management of long-term dependencies. RNNs have been successfully applied to learn features from SMILES, improving performance of drug discovery.[52,53] In addition, numerous studies proposed the advanced recurrent neural networks and integrated deep reinforcement learning for *de novo* molecular generation.[54–56]

## S5. Graph neural networks

DNNs, CNNs, and RNNs have been widely applied to various fields. However, they can only handle Euclidean structured data like images (2D grids) and text (1D sequences). They are unable to process graph-structured data due to the relationships among nodes, the absence of strict order among nodes, and the different numbers of neighbors for each node. Nevertheless, in the real world, there is a substantial amount of graph-structured data, such as social networks, transportation networks, and protein-protein interaction networks. Therefore, graph neural networks (GNNs) are specifically designed for graph-structured data and have achieved great progress.[57] GNNs can be further divided into spectral methods and spatial methods.[58]

**Spectral-based graph neural networks:** This type of GNNs designs graph convolutions by referencing to filters in signal processing.[59] Specifically, graph signal is mapped into the spectral space via Fourier transform, and then convolution operations are performed in the spectral space. Finally, the signal is mapped into graph domain by Fourier inverse transform.[59] Spectral-based GNNs, such as SCNN,[60] ChebNet,[61] and GCN,[62] have the strong theoretical foundation. In particular, GCN is one of most popular models in graph deep learning. Traditional spectral methods lead to higher computational complexity because they rely on the eigendecomposition of graph Laplacian matrix. However, GCNs simplify this process by using a first-order approximation of Chebyshev spectral convolutions, thus reducing computational complexity and improving efficiency.

**Spatial-based graph neural networks:** Spatial-based GNNs directly design graph convolutions by information propagation based on graph structure. The key points of spatial-based GNNs are message passing and neighbor aggregation.[63] In other words, spatial-based GNNs generate node representation by using specific aggregation function to fuse messages from node itself and neighbors. Based on different aggregation functions, one line of spatial-based GNN methods, such as GraphSAGE,[64] GAT,[65] and GIN,[66] have been developed. Among these models, GraphSAGE promoted the application of GNNs on large-scale graphs by randomly selecting the fixed-size neighbors to aggregate information, instead of using the full neighbor sets. In addition, GraphSAGE proposed three aggregator functions including mean operation, LSTM operation, and pooling operation. Inspired by GraphSAGE, GAT proposed a mask attention-based aggregation function to improve the representation ability. The masked attention models focus only on learning the importance coefficients between first-order neighbors and each target node. Similar to Transformer,[67] GAT also adopts multi-head attention mechanisms to increase the stability of model. Each masked attention model is independently executed, and then representations from multiple attention models are fused as the final representation of nodes.

Compared to spectral methods, spatial methods are more flexible and general. Because spatial methods can design different aggregation functions for various graph data. However, spatial methods are difficult to capture the global topology in graph data. In contrast, spectral graph neural networks define the convolutional operations by leveraging spectral graph theory, thus formulating a solid theoretical foundation. Spectral methods rely on the eigendecomposition of graph Laplacian matrix, which is conducive to capture global information, but limits their scalability for large or dynamic graphs[68]. Interestingly, the convolution formula of GCN suggests that it generates node representations by aggregating messages from node itself and neighbors. Therefore, GCN can be also treated as a spatial-based method, and relieve the gap between spatial- and spectral-based approaches. In addition, spectral or spatial graph neural networks suffer from over-smoothing phenomenon when the number of hidden layers increases[69,70]. Therefore, regularization[70,71] and residual connection[72,73] techniques are used to mitigate the over-smoothing phenomenon in deep graph neural networks.

Spectral and spatial graph neural networks have emerged as a leading paradigm for 2D graph data. Nevertheless, unlike 2D graph data, geometric graphs, such as 3D molecular conformation and protein structures, exhibit symmetries of translations, rotations, and/or reflections, which pose significant challenges to the direct application of conventional graph neural networks[74]. Therefore, various geometric GNNs with invariant or equivariant properties extend the message passing mechanism to geometric

graphs. In invariant GNNs, 3D coordinates of nodes are transformed into geometric invariant scalars, such as relative distances between nodes, and dihedral angles. These scalars and node features are updated and integrated to generate the output representation by using message passing mechanism[75,76]. In contrast to invariant GNNs, equivariant GNNs simultaneously update both invariant features and equivariant features to capture the geometric consistency in 3D graphs[77]. Previous works demonstrate that performance of equivariant GNNs is superior to invariant GNNs for geometric graphs[74,78].

In addition, as a matter of fact, most graphs in the real world often contain multiple types of nodes or edges, known as heterogeneous networks. For example, most biomedical networks integrate multiple types of entities (e.g., genes, proteins, drugs, and pathways) and relationships (e.g., drug-drug interaction, drug-target interaction, protein-protein interaction). Compared to homogeneous networks, heterogeneous networks contain more abundant semantic information and heterogeneity[79]. However, these semantics are ignored in traditional graph neural networks. Therefore, there is an interest exploring heterogeneous graph neural networks in which design specific aggregate functions to encode topological structure and semantic information between nodes, generating high-quality representation[80,81]. In particular, biomedical heterogeneous networks can simulate the regulatory relationship between biological entities in life systems. Therefore, heterogeneous graph neural networks have become more popular in the field of drug discovery, achieving better performance[82,83].

GNNs have been used to learn representations from molecular graphs for drug discovery. D-MPNN[84] designed a directed message passing neural network on molecular graphs and incorporated global features from RDKit[85] to enhance molecular property prediction. Attentive FP[86] and AMPNN[87] further improved prediction performance by integrating graph attention mechanisms. Recently, GeoGNN[88] captured local and global spatial structures of molecules by predicting bond lengths, angles, and atomic distance matrices for understanding molecular properties. Interestingly, the integration of reinforcement learning with GNNs has improved the performance of drug design. GCPN,[89] MolDQN,[90] DeepGraphMolGen,[91] and MNCE-RL[92] combine the strengths of GNNs and reinforcement learning to push the boundaries of targeted molecule generation.

## S6. Transformer

Transformer[67] is a multi-head attention-based sequence-to-sequence model that adopts an encoder-decoder framework. The encoder aims to map the sequential data into low-dimensional representations; the decoder maps these representation vectors to specific outputs. **Encoder:** The encoder is composed of multiple modules with the identical structure. Each module contains a multi-head self-attention layer and a fully connected feed-forward network layer that consists primarily of two linear layers with a non-linear function. Each sub-layer internally executes residual connections[42] and layer normalization[93] operations that can accelerate the convergence speed of models. The self-attention mechanisms primarily learn the long-range dependencies among elements (e.g., tokens) in sequential data.[67] In self-attention models, the sequential data is mapped into three matrices (i.e., query, key, and value) by linear transformations.[94] Subsequently, the attention score is calculated by the dot product operation of the query with the key, and then normalized via the scaling factor and Softmax function. The normalized attention score denotes the associated coefficient among terms in sequential data. Therefore, self-attention output is obtained by the weighted summation for the value where all weighted coefficients are extracted from the attention scores. In addition, due to the self-attention mechanism without the ability capturing positional information, it cannot distinguish the identical elements at different positions within a sequence. Therefore, in Transformer, sine and cosine-based positional functions are designed at the bottoms of encoder and decoder to calculate absolute positions of each element in sequences. However, the absolute position encodings generate the poor performance when the length of sequence is over the given thresholds. Being aware of this issue, various advanced methods, such as relative positional encodings[95], and rotary position embeddings (RoPE)[96], are integrated into Transformer or their variants, and have been applied to drug discovery.

**Decoder:** Similar to encoder, the decoder is also composed of a stack of multiple identical modules that mainly consist of multi-head attention layers and feed-forward network layers. In addition, the decoder proposes masked multi-head attention layers to prevent current positions from attending to subsequent positions. In other words, the masked attentions ensure that the representation at each position depends only on the information from the left part of current position. This is mainly because the output of each position only needs to consider the influence of the known data from the left side of current position. Finally, outputs are completed by the learnable linear transformation and Softmax function.

In Transformer, multi-head self-attention models are employed to increase the stability. To be specific, the data of each layer is projected into multiple query, key, and value matrices in different sub-spaces, respectively. Then, self-attention operations are performed independently in each sub-space. Finally, the outputs of self-attention in each sub-space are fused as the final representation. The self-attention mechanisms in different sub-spaces can capture multi-view correlation within sequential data. Therefore, multi-head self-attention models improve the representational capacity and stability of Transformer.

## S7. Vision and graph Transformers

Inspired by the success of Transformer in natural language processing, Vision Transformer (ViT)[97] and Graph Transformer[98] are developed to encode vision images and graph data, respectively.

**Vision Transformer:** ViT is a variant of Transformer on image data,[97] and remains the core principles of original Transformer. There are four key components in ViT. *Patch embedding:* ViT first divides the input image into fixed-size patches, which are then linearly embedded into a sequence of vectors. *Positional encoding:* Similar to Transformer, positional encodings are added into the patch embeddings to provide model with the spatial arrangement of each image patch. *Transformer encoder:* The core of ViT is a standard Transformer encoder with minor variations like the placement of nonlinear activation functions. *Prediction head*: Inspired by the *[CLS]* token in BERT,[94] ViT incorporates a learnable embedding for the sequence of patch embeddings at the output of Transformer encoder. This learnable embedding can effectively encode the features of images, and is mapped into a classification score by multilayer perceptrons. ViT has shown promising results on various benchmark datasets, rivaling or even surpassing the performance of CNN-based models on computer vision tasks. ViT exhibits advantages that include scalability to larger image sizes, and easy parallelization. In addition, ViT can capture long-range dependencies among image patches in full images, which can be beneficial for understanding complex visual scenes.

**Graph Transformer:** Similar to ViT, Transformer has been extended to graph structure data. In particular, a graph Transformer named Graphormer[98] has achieved competitive performance against GNNs. To leverage the topology of graphs, Graphormer incorporates *Centrality Encoding, Spatial Encoding*, and *Edge Encoding* into Transformer. These encoding methods ensure that Transformer can learn the topology information of graphs. *Centrality encoding:* Node centrality can measure the importance of each node in a graph. Therefore, Graphormer proposed centrality encoding to enhance the importance signal of nodes. To be specific, each node is assigned two learnable embedding vectors according to its indegree and outdegree. These two vectors are added to node features. *Spatial encoding:* It aims to encode the topology structure of graphs. Graphormer assigns a learnable scalar based on the shortest path distance among nodes, if two nodes are connected. If not, the scalar is set to a special value. The learnable scalar is treated as a bias term in self-attention module and shared across all layers. *Edge Encoding:* Edge features may contain additional structural information,[99] such as the type of bond between two atoms in a molecular graph. Therefore, Graphormer developed the edge encoding to better inject edge features into attention mechanism. For each node pair, Graphormer computes an average of the dot-product of the edge feature and a learnable weight embedding along the shortest path, and then the average value is treated as a bias term in the attention module. In the light of Graphormer, graph Transformer has attained more and more attention and emerged as a promising paradigm for graph data. The key advantage of graph Transformer lies in its ability to capture the relevance among nodes and enhancing the topology structure in graphs, such as node centrality, spatial distance, and edge features.

## S8. Unimodal pre-training tasks

### Perturbation contrastive learning

It is one of the earlier self-supervised learning paradigms, such as SimCLR,[100] GraphCL,[101] and GCC,[102] which focus on single modality data. The underlying principle of contrastive learning is to maximize agreement between positive samples, and minimize agreement between negative samples.[100,103] In contrastive learning, the agreement between samples is usually measured by the mutual information. There are typical mutual information estimators including Donsker-Varadhan estimator,[104] Jensen-Shannon estimator,[105] and noise-contrastive estimation (InfoNCE).[106] In particular, InfoNCE is commonly used as an objective of contrastive learning for drug discovery. Perturbation contrast focuses on learning representations by contrasting instances from different views or augmentations. To be specific, a given object as anchor, the augmentation samples are generated by various perturbations, such as randomly dropping some edges or nodes in a graph, and randomly rotating or cropping images. The augmented instances from the same anchors are treated as positive examples, while instances from different anchors are served as negative examples. Perturbation contrast learning-based drug discovery methods, such as CSGNN[107] and MolCLR,[108] have achieved progress. MolCLR[108] designed three molecule graph augmentations including atom masking, bond deletion, and subgraph removal in molecular graphs. Perturbed molecule graphs from the same molecule are denoted as positive pairs, whereas those from different molecules are denoted as negative pairs. Subsequently, the graph neural network-based encoders are pre-trained by using the normalized temperature-scaled cross-entropy contrastive loss[109] that is a specific type of InfoNCE loss.

Unfortunately, these methods employ random perturbations, damaging the original biological semantics behind molecular data[101,110]. For example, for a molecular graph, minor perturbations, such as removing a chemical atom or bond, may drastically change their properties or semantics. In addition, perturbation contrastive learning models mainly focus on graph structures, ignoring fundamental domain knowledge within molecules[111]. Therefore, more and more studies including MoCL,[112] KCL,[110] CKGNN,[113] and KANO,[114] use the domain knowledge for data augmentations, and then use contrastive learning to pre-train encoders. The knowledge-based contrastive training methods can capture features behind molecule data and biochemical

knowledge, thus further improving the performance of drug discovery.

### Masked prediction

The pre-training task, initially developed for natural language processing by BERT,[94] has emerged as a leading pre-training paradigm. The masked prediction task can improve the model's ability to understand the internal features of input data. In the masked language model proposed by BERT, tokens in input sequences are randomly perturbed by specific token <Mask>, and then encourage Transformer encoder to reconstruct these masked tokens based on context. With the success of BERT, masked language model and its variants, such as RoBERTa,[115] T5,[116] and SpanBERT[117] have been applied to computer vision[118,119] and drug discovery.[120,121] In drug discovery, masked prediction is initially used to encode 1D sequences that are treated as sentences of the language of chemistry.[121,122] Subsequently, masked language models are extended to molecular graph data.[123,124]

### Autoregressive prediction

It is initially proposed in natural language models including GPT,[125] Transformer-XL,[126] and PaLM.[127] Specifically, autoregressive models commonly use Transformer or its decoders to predict next token based on the preceding tokens in sequence data. It is worth noting that autoregressive pre-training tasks are often integrated with decoder models. In the field of drug discovery, 1D sequences are often treated as the chemical language systems. Therefore, it is natural to develop GPT-like chemical language models for molecule generation.

## S9. Comparison unimodal and multimodal pre-training tasks

Compared to unimodal pre-training methods, multimodal approaches can gain an indepth and systemic understanding to physicochemical space by learning consistency and complementarity among different modalities. On the other hand, multimodal pre-training models can integrate molecular structure, attribute, and biological effect in living systems to improve robustness and generalization in multiple drug discovery tasks. For example, a structure-based pre-training model may be able to achieve competitive performance in molecular property prediction, but fail to precisely predict drug-target interactions to a great extent. This is mainly because the molecular property is often closely related to its own structure[63], while the complex regulatory relationships inside living system play a more important role in drug-target interaction prediction.

However, multimodal pre-training models for drug discovery are full of more challenges compared to unimodal approaches. First, it is difficult to align multiple modalities in real drug discovery. Because there are great scenarios that many samples are lack of some modality of data, limiting the performance of multimodal pre-training models. Second, multimodal pre-training models often integrate multiple encoders and fusion mechanisms, resulting in large-scale parameters. Therefore, multimodal pre-training models exhibit a higher computational complexity than unimodal approaches. In addition, there are more complex mechanisms behind multiple modalities of data. Therefore, how to discriminate and fuse consistency and complementarity among various modalities is significant to improve the performance of drug discovery[128].

## S10. Cross-modal contrastive learning

It is more suitable for multimodal pre-training paradigms, and aims to map data of different modalities to a common space. There are similar principles and loss functions between perturbation and cross-modal contrastive learning. However, in cross-modal contrastive learning, the instances of the same object in different modalities are treated as positive example pairs, and the samples of different objects are treated as negative pairs.[129,130] Initially, CLIP[131] proposed an image-text contrastive learning model that has been integrated into various multimodal pre-training methods, such as VLMO,[132] BLIP.[133] Inspired by CLIP, numerous studies have attempted to jointly train graph encoders and SMILES encoders by a contrastive learning objective for molecular property prediction.[134,135] To further improve performance, more and more works train GNNs by maximizing the mutual information between the embedding of 2D molecular graph and 3D molecular geometry.[136,137] In addition, a new trend is to integrate molecular structure (e.g., SMILES and 2D graphs) and scientific text for drug discovery.[138,139]

## S11. Multi-modal matching prediction

Multi-modal matching prediction aims to learn high-quality representation by predicting whether a pair of samples from two modalities are matched (positive pair) or not matched (negative pair).[140] Similar to cross-modal contrastive learning, two samples of the same object in different modalities are regarded as positive example pairs, while all samples from different objects are served as negative pairs. However, a key difference between matching prediction and contrastive learning is that the former maximizes mutual information of positive pairs and minimizes one of negative pairs, while the latter aims to categorize all samples into positive or negative classes. Therefore, matching prediction is a binary classification task, and often

employs binary cross-entropy function as loss function. Matching prediction can guide models to learn the correlation between different modalities. Therefore, matching prediction can enhance the performance of multimodal learning tasks, and has been widely used for various multimodal pre-training fields. In particular, Transformer-based image-text matching prediction was first proposed by UNITER[141] and has been added into vision-and-language representation learning algorithms to improve downstream tasks.[142] In order to find more informative negatives, ALBEF[143] proposed a contrastive similarity-based hard negative mining strategy for image-text matching tasks. To be specific, for each image in a mini-batch, texts in the same batch which are more similar to the image, have a higher chance to be selected as negative samples. In ALBEF, matching prediction can be treated as the complement of contrastive learning. Inspired by image-text matching prediction, ISMol[144] proposed molecule image-SMILES matching-based pre-training models for molecular property prediction. MolCA[145] utilized both 2D molecule-text matching prediction and contrastive learning to train the graph encoder and the cross-modal projector.

## S12. Multi-modal masked prediction

Recently, masked prediction tasks have been used for multimodal molecular representation learning, where these models predict masked information based on prompts of other modalities.[144] This requires models to understand the complex relationship and interaction between different modalities, further promoting the alignment of multimodal data. Inspired by BERT, a large volume of studies used the SMILES to replace the molecule names in biomedical text. Subsequently, based on these wrapped sequences, the encoders are pre-trained via masked language model for drug discovery.[146,147] In addition, following the ideas of deepwalk,[148] deepR2cov[149] and BioERP[150] use a random walk strategy guided by meta paths to generate node sequences that are treated as the special "sentences" describing semantic within biomedical heterogeneous networks. Next, these node sequences are randomly masked, and fed into a Transformer encoder to predict the masked entities, generating the low-dimensional representations for drug-target interaction prediction.

## S13. Multi-modal autoregressive prediction

In multi-modal autoregressive prediction, the data of different modalities is integrated into a unified sequence in which a portion of modalities serves as the conditions or prompts for the rest of modalities. Subsequently, autoregressive pre-training tasks predict next tokens based on the preceding tokens in multimodal sequences, preventing positions from attending to subsequent positions. For drug discovery, multiple studies developed a unified autoregressive model of text and molecules via the wrapped sequences.[151] This type of cross-modal autoregressive approach can promote the fine-grained understanding for molecular contexts, thus improving the performance of drug discovery. On the other hand, autoregressive pre-training technologies have been extended to graph data including molecular graphs.[152,153]

## S14. Joint training

In joint training scheme, the encoder is trained via combining self-supervised tasks and downstream tasks. To be specific, the loss value is a weighted sum of self-supervised loss and downstream loss. Therefore, the joint training mechanism is treated as a multi-task learning paradigm, in which a trade-off hyperparameter controls the contributions of pre-training and downstream tasks. Generally, the weight value of pre-training tasks is smaller than that of downstream tasks. Therefore, the pre-training tasks are served as a regularization or auxiliary term of downstream tasks.[154] The joint strategies have been used for training self-supervised tasks and drug discovery tasks.[155,156]

## S15. Unsupervised representation learning

In unsupervised representation learning, an encoder and a self-supervised predictor are trained by pre-training tasks, and then the encoder is frozen and added to the front of a new predictor for downstream tasks. In other words, the encoder learns the low-dimensional representations, and then these representations are directly fed into a new predictor for downstream tasks. Therefore, in unsupervised representation learning, the pre-training phase can be regarded as the feature extraction process. Initially, most methods proposed pre-training models to generate low-dimensional representation, and then used fully-connected neural networks or machine learning for drug discovery.[149,150,157]

## S16. Two-stage training

Similar to unsupervised representation learning, encoders are trained by self-supervised tasks, thus obtaining an encoder with high-quality parameters. Subsequently, the pre-trained encoder is served as the initial model in the fine-tuning stage, and fine-tuned together with a prediction head of downstream tasks. In two-stage training, the pre-training process can be considered as the parameter initialization of encoders. However, with the continuous growth of pre-training model parameters,

full parameter fine-tuning methods generate the tremendous computational cost. Simultaneously, the models may suffer from catastrophic forgetting problems when all pre-trained parameters are optimized to meet requirements of downstream tasks. Therefore, parameter efficient fine-tuning techniques are proposed in natural language processing tasks and applied to drug discovery. Parameter efficient fine-tuning algorithms aim to reduce the size of fine-tuning parameters while achieving comparable performance to full fine-tuning methods. In parameter efficient fine-tuning algorithms, most or all parameters are frozen, thus relieving catastrophic forgetting problems and over-fitting problems[158, 159]. The parameter efficient fine-tuning algorithms can be categorized into **additive, selective, and reparameterized fine-tuning** methods.[158, 159] **Additive fine-tuning:** This method freezes pre-training encoders and introduces the small-scale parameters for each downstream task. Adapter[160] and Soft Prompt[161, 162] are the most popular additive fine-tuning algorithms. KANO[114] proposed knowledge-enhanced functional prompt learning for molecule property prediction. **Selective fine-tuning:** It freezes the majority of parameters, but selects a subset of pre-trained parameters during fine-tuning on downstream tasks, thus realizing parameter efficient fine-tuning. BitFit[163] only updated the bias terms and the task-specific predictors while freezing the rest of parameters. **Reparameterized fine-tuning:** This method hypothesizes that change of weight parameters has a low intrinsic rank during model adaptation.[164] Therefore, LoRA[164] freezes the pre-trained parameters and injected trainable low-rank decomposition matrices into each layer of Transformer for downstream task-specific fine-tuning. LoRA and its derivatives have become one of most popular parameter efficient fine-tuning methods. However, the reparameterized fine-tuning is still an emerging technique in the field of drug discovery.

## S17. Application of multimodal pre-training models in drug discovery

The developments of multimodal pre-training techniques provide opportunities to drug discovery, including molecular generation, molecular property prediction, drug-drug interaction prediction, drug-target interaction prediction, and molecule captioning.

### Molecular generation

Molecular generation aims to design new drugs with specific properties that include high affinity, safety and activity, low toxicity, and structural novelty. In recent years, molecular generation is benefit from multimodal pre-training advances. TamGen[165] proposed a GPT-like chemical language model for target-aware molecule generation, in which Transformer decoder is pre-trained by autoregressively predicting next SMILES tokens, and fine-tuned by integrating 2D molecular graphs. Lingo3DMol[166] proposed an autoregressive pre-training approach that aims to reconstruct the perturbed molecule back to its original state in both 2D and 3D representations. In fine-tuning stage, the three encoder layers were fixed. The 2D topology decoder generated sequence fragments and local coordinates, and other decoders generated 3D coordinates. 3DSMILES-GPT[167] treated both SMILES and atomic 3D coordinates as linguistic expressions, and then used autoregressive prediction tasks to train a Transformer-like decoder. In fine-tuning stage, 3DSMILES-GPT integrated surface atomic coordinates from pockets for 3D molecular generation, and employed reinforcement learning to optimize the biophysical and chemical properties of the generated molecules.

In addition, with the success of pre-training larger models in natural language processing, biomedical text-based molecular generation also has achieved significant progress. Based on corpus consisting of SMILES sequence and textual description, MolT5[168] utilized the recovering masked spans that are the extension of masked language models, to pre-train Transformer-based T5.[116] The pre-training model is fine-tuned for molecule captioning and generation. Analogously, Ada-T5,[169] ChatMol,[170] Text+Chem T5,[171] and nach0[172] pre-trained Transformer on textual description and SMILES to incorporate chemical and linguistic knowledge. Inspired by MolT5, 3D-MolT5[18] mapped the fine-grained 3D structure-aware fingerprint to a specialized 3D token. The 3D structure tokens enable the seamless combination of molecular sequence and 3D structure in a tokenized format. Next, 3D-MolT5 encoded molecular sequence, 3D structure, and text sequences within T5-based unified architecture via recovering masked spans and cross-modal translation tasks. The pre-training model is fine-tuned for molecular property prediction, molecule captioning and generation. Recently, GIT-Mol[173] integrated graph isomorphism network,[66] SciBERT,[174] and Swin Transformer[175] to encode SMILES and 2D molecule graphs, molecular images and captions. In the pre-training phase, GIT-Mol employed cross-modal contrastive learning and matching prediction. The pre-training model is prompt-tuned for molecule captioning and generation.

### Molecular property prediction

Molecular property prediction, a fundamental task in drug discovery, aims to infer the molecular attributes based on structure features. Multimodal pre-training techniques have been developed for molecular property prediction. MM-Deacon[176] leveraged dual separate Transformers to encode SMILES and International Union of Pure and Applied Chemistry (IUPAC) via contrastive learning. The task-specific prediction layer was attached to the pre-trained model for drug discovery. DVMP[177] proposed a dual-view molecular pre-training method, combining SMILES and 2D molecular graphs. To be specific, SMILES and

2D graph of molecules are randomly masked several elements, and then fed into a Transformer and a GNN, respectively. DVMP proposed two pre-training objectives including the maximizing the semantic consistency and reconstructing masked elements. The pre-trained Transformers or GNNs are used for molecular property prediction. GRAPHMVP[178] encoded 2D graphs and 3D geometric structures by graph isomorphism network and SchNet.[179] Concurrently, the correspondence and consistency between 2D graphs and 3D geometric structures are unified by cross-modality contrastive learning and variational representation reconstruction. U2-3DPT[180] learned 2D and 3D molecular representations by using reconstruction of masked atom and 3D coordinates, 3D conformation generation conditioned on 2D graph, and 2D graph generation conditioned on 3D conformation. To improve molecular property prediction, GeomGCL[137] designed a geometric contrastive learning and dual geometric message passing networks to adaptively learn the 2D and 3D structure. Similarly, 3D Infomax[136] trained 2D and 3D structure-based dual-channel GNNs by contrastive learning. However, it is worth noting that only 2D graph-based neural networks were fine-tuned for molecular property predictions. ISMol[144] utilized respectively a visual Transformer and ChemBERTa to encode molecular image and SMILES string. Simultaneously, In ISMol, cross-attention model is used for information interaction between molecular image and SMILES. In addition, ISMol is pre-trained by three self-supervised tasks, i.e., image-SMILES matching prediction, SMILES mask reconstruction and fingerprint class prediction. MGIB[181] observed molecular graphs from atom view and motif view, and extended the graph information bottleneck into the contrastive learning framework for molecular property prediction. Interestingly, MOLEBLEND[182] introduced a pre-training model via modality blending for property prediction. To be specific, MOLEBLEND constructed a modality-blended atom relation matrix. Similar to Graphormer,[98] the relation matrix was injected into self-attention modules for unified cross-modality encoding. Subsequently, MOLEBLEND recovered modality-specific atom relations. To integrate more modalities, MOCO[135] utilized SMILES strings, fingerprints, 2D graphs, and 3D geometries to drive four accompanying neural networks, and then leveraged an attention network to fuse multimodal embedding as a final representation. MOCO was pre-trained via a contrastive objective that maximized the consistency between the representation of each modality and the final representation, thus improving the performance of molecular property prediction. KANO[114] utilized Periodic Table to construct a knowledge graph, and then designed a knowledge graph and 2D molecular structure-based contrastive learning to explore microscopic atomic relations. Further, KANO proposed a functional prompt-based fine-tuning method to improve molecular property prediction.

On the other hand, molecular structure and text-based pre-training models are applied to molecular property prediction. KV-PLM[146] proposed a deep learning system that bridges molecular structures and biomedical texts. In KV-PLM, the molecular entities in biomedical texts are replaced with the segmented SMILES. KV-PLM was pre-trained via masked language model to capture the meta-knowledge between different semantic units, and then fine-tuned for molecular property prediction, named entity recognition, and relation extraction. Inspired by KV-PLM, MolLM[183] proposed a unified language model that integrated biomedical texts with 2D and 3D molecular representation. MolLM initialized text encoder with checkpoint of KV-PLM,[146] and employed graph Transformer to encode both 2D molecular graph and 3D geometric structures. Finally, encoders are pre-trained via inter- and intra-modal contrastive learning, and then fine-tuned for molecular property prediction, molecule captioning, and editing. MoleculeSTM utilized MegaMolBART,[184] GraphMVP,[185] and SciBERT[174] to encode SMILES, 2D molecule graph, and textual description, and then these encoders are pre-trained via cross-modal contrastive learning. The pre-training models are fine-tuned for molecular property prediction, molecule editing and structure–text retrieval. MoMu[186] proposed a similar multimodal model for drug discovery.

## Drug-drug interaction prediction

Drugs may interact with each other when using drug combinations to treat diseases. Drug–drug interaction (DDI) increases the risk of adverse drug reactions. Therefore, it is significant to explore multimodal self-supervised learning-based DDI predictions. MIRACLE[157] proposed multi-view graph contrastive representation learning for drug-drug interaction prediction, where 2D molecular graphs and DDI networks were encoded by bond-aware message passing networks and graph convolutional networks,[62] respectively. MRCGNN[155] proposed multi-relational contrastive learning to jointly train graph neural networks that hierarchically integrated 2D molecular graph and DDI networks. Based on the contrastive learning perspective, TIGER[156] introduced a relation-aware heterogeneous graph Transformer to capture multiple relationships within molecular graph and interaction networks for DDI prediction. HS-GPF[187] constructed drug-motif interaction graphs and hierarchical pre-training tasks to train backbone graph neural networks, thus capturing structural and relational insights. In HS-GPF, a graph prompt learning framework is specially designed to integrate pre-training tasks and DDI prediction into a uniform format. Similarly, H2D[83] proposed a hierarchical and heterogeneous graph learning framework for DDI prediction where learning the shared information between molecular structure and biomedical networks via contrastive learning. To improve DDI prediction, HetDDI[82] synthesized 2D graph and rich semantic information in biomedical interaction networks via attribute masking and link prediction tasks.

### Drug-target interaction prediction

Drug-target interaction (DTI) prediction aims to determine whether a given drug and target can interact with each other. Generally, DTI prediction is also treated as a drug repositioning strategy that aims to screen potential drugs from existing ones for given targets.[188] CSCo-DTA[189] learned features from molecular graphs and interaction networks via cross-modal graph contrastive learning approaches. Subsequently, CSCo-DTA used joint training methods to integrate contrastive learning and drug–target affinity prediction that is a similar task to DTI prediction. Based on SMILES and 2D molecular graphs, DrugLAMP[190] respectively employed mask prediction and contrastive learning to train Transformers and graph convolutional networks for drug–target interaction prediction. On the other hand, BioT5[147] integrates molecular SELFIES,[191] protein sequences, general texts, and wrapped texts[151] where molecule names were replaced with their corresponding SELFIES and gene names were appended with related protein sequences. To reduce the gap between pre-training and downstream tasks, BioT5 adopted the prompt-based fine-tuning for drug-target interaction prediction, and molecule property prediction.

### Molecule captioning

Given a molecular structure, molecule captioning aims to provide comprehensive text descriptions, enhancing the understanding of key characteristics that include molecule name, chemical formula, basic structure, properties and functions.[192] Compared with other tasks in drug discovery, molecule captioning is still an emerging field. With the successful application of pre-trained large models in natural language and computer vision, there is increasing interest in deep learning-based molecule captioning. MolCA[193] employed GNN and Galactica model[194] to encode 2D molecular graphs and textual description. The molecular graph-language encoders are pre-trained via contrastive learning and matching prediction. Finally, MolCA employed uni-modal adapter for molecule captioning, IUPAC name prediction, and molecule-text retrieval. Inspired by MolCA, 3D-MOLM[195] proposed the 3D molecule-text projector to enables Llama2[196] to interpret and analyze 3D molecules. MolFM[197] proposed multi-channel encoders including 2D molecule structure-based graph isomorphism networks,[66] biomedical text-based MolT5, and knowledge graph-based TransE,[198] and then the multi-channel encoders were pre-trained via structure-text contrastive learning, cross-modal matching prediction, masked language modeling and knowledge graph reconstruction. In MolFM, the representations from pre-trained graph isomorphism networks and MolT5 encoders were fused, and then fed into MolT5 decoder to generate molecule captioning. UniMoT[199] introduced a vector quantization-driven tokenizer that incorporated a causal Q-Former[200] to bridge the gap between molecules and texts. The causal Q-Former was pre-trained via molecule-text contrastive learning, matching prediction, and fine-tuned via prompt learning for molecule captioning. In addition, we found that molecule captioning and text-based molecule generation are often treated as bi-directional translation between molecules and language.

## S18. Summary of all cited methods

Here, we summarize the molecular modalities, self-supervised tasks, and downstream tasks of the cited methods.

**Table S2** Key description of all cited methods

| Methods | Modalities | Self-supervised Task | Drug Discovery |
|---|---|---|---|
| REINVENT | 1D Sequence | Autoregressive | Molecular Generation |
| ChemTS | 1D Sequence | Autoregressive | |
| GCPN | 2D Graph | None | |
| MNCE-RL | 2D Graph | None | |
| MolGPT | 1D Sequence | Autoregressive | |
| TamGen | 1D Sequence, 3D Geometry | Autoregressive | |
| Lingo3DMol | 1D Sequence, 2D Graph | Autoregressive | |
| 3DSMILES-GPT | 1D Sequence, 3D Geometry | Autoregressive | |
| MolT5 | Molecule Caption, 1D Sequence | Masked Prediction | |
| Ada-T5 | Molecule Caption, 1D Sequence | Masked Prediction | |
| ChatMol | Molecule Caption, 1D Sequence | Masked Prediction | |
| Text+Chem T5 | Molecule Caption, 1D Sequence | Masked Prediction | |
| nach0 | Molecule Caption, 1D Sequence | Masked Prediction | |

**Table S2** Summary of all cited methods

| Methods | Modalities | Self-supervised Task | Drug Discovery |
|---|---|---|---|
| 3D-MolT5 | Molecule Caption, 1D Sequence, 3D Geometry | Masked prediction, Autoregressive | |
| GIT-Mol | 1D Sequence, 2D Graph, Molecule Caption | Contrastive Learning, Matching Prediction | |
| D-MPNN | 2D Graph | None | Molecular Property Prediction |
| GeoGNN | 2D Graph, 3D Geometry | Masked Prediction | |
| enn-s2s | 2D Graph | None | |
| MAT | 2D Graph | Masked Prediction | |
| Transformer-M | 2D Graph, 3D Geometry | Masked Prediction | |
| SMICLR | 1D Sequence, 3D Geometry, 2D Graph | Masked Prediction, Contrastive Learning | |
| SMILES-BERT | 1D Sequence | Masked Prediction | |
| ChemBERTa | 1D Sequence | Masked Prediction | |
| Pre-GNN | 2D Graph | Masked Prediction, Contrastive Learning | |
| GROVER | 2D Graph | Masked Prediction | |
| MoLFORMER | 1D Sequence | Masked Prediction | |
| MolXPT | 1D Sequence, Molecule Caption | Autoregressive | |
| MGSSL | 2D Graph | Masked Prediction, Autoregressive | |
| DVMP | 1D Sequence, 2D Graph | Masked Prediction | |
| MM-Deacon | 1D Sequence, 2D Sequence | Contrastive Learning | |
| GRAPHMVP | 2D Graph, 3D Geometry | Contrastive Learning, Masked Prediction | |
| U2-3DPT | 2D Graph, 3D Geometry | Masked Prediction | |
| GeomGCL | 2D Graph, 3D Geometry | Contrastive Learning | |
| 3D Infomax | 2D Graph, 3D Geometry | Contrastive Learning | |
| MGIB | 2D graph, Interaction Network | Contrastive Learning | |
| MOLEBLEND | 2D Graph, 3D Geometry | Masked Prediction | |
| MOCO | Fingerprints, 1D Sequence, 2D Graph, 3D Geometry | Contrastive Learning, Masked Prediction | |
| KANO | 2D Graph, Interaction Network | Contrastive Learning | |
| KV-PLM | Molecule Captions, 1D Sequence | Masked Prediction | |
| MolLM | Molecule Caption, 1D Sequence, 2D Graph, 3D Geometry | Contrastive Learning | |
| MoleculeSTM | Molecule Caption, 1D Sequence, 2D Graph | Contrastive Learning | |
| MoMu | Molecule Caption, 2D Graph | Contrastive Learning | |
| DeepDDI | Fingerprints | None | Drug-drug Interaction Prediction |
| DeepSynergy | Fingerprints | None | |
| MIRACLE | 2D Graph, Interaction Network | Contrastive Learning | |
| MRCGNN | 2D Graph, Interaction Network | Contrastive Learning | |
| TIGER | 2D Graph, Interaction Network | Contrastive Learning | |
| HS-GPF | 2D Graph, Interaction Network | Contrastive Learning | |
| H2D | 2D Graph, Interaction Network | Contrastive Learning | |
| HetDDI | 2D Graph, Interaction Network | Masked Prediction | |
| DeepDTA | 1D Sequence | None | |
| MATT_DTI | 1D Sequence | None | |
| DEEPScreen | 2D Graph | None | |

**Table S2** Summary of all cited methods

| Methods | Modalities | Self-supervised Task | Drug Discovery |
|---|---|---|---|
| MSSL2drug | Interaction Network | Masked Prediction, Contrastive Learning | Drug-target Interaction Prediction |
| Transfor-merCPI2.0 | 2D Graph | Masked Prediction | |
| Interformer | 3D Geometry | Contrastive Learning | |
| deepR2cov | 2D Graph, Interaction Network | Masked Prediction | |
| BioERP | Interaction Network | Masked Prediction | |
| CSCo-DTA | 2D Graph, Interaction Network | Contrastive Learning | |
| DrugLAMP | 1D Sequence, 2D Graph | Masked Prediction | |
| BioT5 | 1D Sequence, Molecule Caption | Masked Prediction | |
| MolCA | 2D Graph, Molecule Caption | Contrastive Learning, Matching Prediction | Molecule Captioning |
| 3D-MOLM | Molecule Caption, 3D Geometry | Matching Prediction, Contrastive Learning, Autoregressive | |
| MolFM | 2D Graph, Molecule Caption, Interaction Network | Contrastive Learning, Matching Prediction, Masked Prediction | |
| UniMoT | Molecule Caption, 1D Sequence, 2D Graph | Contrastive Learning, Matching Prediction, Autoregressive | |

## References


**1.** Wishart, D. S. *et al.* Drugbank 5.0: a major update to the drugbank database for 2018. *Nucleic acids research* **46**, D1074–D1082 (2018).

**2.** Gaulton, A. *et al.* The chembl database in 2017. *Nucleic acids research* **45**, D945–D954 (2017).

**3.** Santos, A. *et al.* A knowledge graph to interpret clinical proteomics data. *Nat. biotechnology* **40**, 692–702 (2022).

**4.** Walsh, B., Mohamed, S. K. & Nováček, V. Biokg: A knowledge graph for relational learning on biological data. In *Proceedings of the 29th ACM International Conference on Information & Knowledge Management*, 3173–3180 (2020).

**5.** Chandak, P., Huang, K. & Zitnik, M. Building a knowledge graph to enable precision medicine. *Sci. Data* **10**, 67 (2023).

**6.** Weininger, D. Smiles, a chemical language and information system. 1. introduction to methodology and encoding rules. *J. chemical information computer sciences* **28**, 31–36 (1988).

**7.** Heller, S. R., McNaught, A., Pletnev, I., Stein, S. & Tchekhovskoi, D. Inchi, the iupac international chemical identifier. *J. cheminformatics* **7**, 1–34 (2015).

**8.** Daylight Chemical Information Systems, I. Smarts-a language for describing molecular patterns. (2019).

**9.** Tang, B. *et al.* A self-attention based message passing neural network for predicting molecular lipophilicity and aqueous solubility. *J. cheminformatics* **12**, 1–9 (2020).

**10.** Capecchi, A., Probst, D. & Reymond, J.-L. One molecular fingerprint to rule them all: drugs, biomolecules, and the metabolome. *J. cheminformatics* **12**, 1–15 (2020).

**11.** Kim, S. *et al.* Pubchem 2019 update: improved access to chemical data. *Nucleic acids research* **47**, D1102–D1109 (2019).

**12.** Durant, J. L., Leland, B. A., Henry, D. R. & Nourse, J. G. Reoptimization of mdl keys for use in drug discovery. *J. chemical information computer sciences* **42**, 1273–1280 (2002).

**13.** Rogers, D. & Hahn, M. Extended-connectivity fingerprints. *J. chemical information modeling* **50**, 742–754 (2010).

**14.** Matter, H. & Pötter, T. Comparing 3d pharmacophore triplets and 2d fingerprints for selecting diverse compound subsets. *J. chemical information computer sciences* **39**, 1211–1225 (1999).

**15.** Liu, Y. *et al.* Spherical message passing for 3d molecular graphs. In *Proceedings of the International Conference on Learning Representations* (2021).

**16.** Grisoni, F., Ballabio, D., Todeschini, R. & Consonni, V. Molecular descriptors for structure–activity applications: a hands-on approach. *Comput. Toxicol. Methods Protoc.* 3–53 (2018).

17. Ryu, J. Y., Kim, H. U. & Lee, S. Y. Deep learning improves prediction of drug–drug and drug–food interactions. *Proc. national academy sciences* **115**, E4304–E4311 (2018).

18. Pei, Q., Wu, L., Gao, K., Zhu, J. & Yan, R. 3d-molt5: Towards unified 3d molecule-text modeling with 3d molecular tokenization. *arXiv preprint arXiv:2406.05797* (2024).

19. Sterling, T. & Irwin, J. J. Zinc 15–ligand discovery for everyone. *J. chemical information modeling* **55**, 2324–2337 (2015).

20. Chen, J., Swamidass, S. J., Dou, Y., Bruand, J. & Baldi, P. Chemdb: a public database of small molecules and related chemoinformatics resources. *Bioinformatics* **21**, 4133–4139 (2005).

21. Kuhn, M., Letunic, I., Jensen, L. J. & Bork, P. The sider database of drugs and side effects. *Nucleic acids research* **44**, D1075–D1079 (2016).

22. Davis, A. P. *et al.* Comparative toxicogenomics database (ctd): update 2021. *Nucleic acids research* **49**, D1138–D1143 (2021).

23. Chen, X., Ji, Z. L. & Chen, Y. Z. Ttd: therapeutic target database. *Nucleic acids research* **30**, 412–415 (2002).

24. Brown, A. S. & Patel, C. J. A standard database for drug repositioning. *Sci. data* **4**, 1–7 (2017).

25. Oughtred, R. *et al.* The biogrid interaction database: 2019 update. *Nucleic acids research* **47**, D529–D541 (2019).

26. Whirl-Carrillo, M. *et al.* Pharmacogenomics knowledge for personalized medicine. *Clin. Pharmacol. & Ther.* **92**, 414–417 (2012).

27. Szklarczyk, D. *et al.* Stitch 5: augmenting protein–chemical interaction networks with tissue and affinity data. *Nucleic acids research* **44**, D380–D384 (2016).

28. Ursu, O. *et al.* Drugcentral: online drug compendium. *Nucleic acids research* gkw993 (2016).

29. Corsello, S. M. *et al.* Discovering the anticancer potential of non-oncology drugs by systematic viability profiling. *Nat. cancer* **1**, 235–248 (2020).

30. Barretina, J. *et al.* The cancer cell line encyclopedia enables predictive modelling of anticancer drug sensitivity. *Nature* **483**, 603–607 (2012).

31. Yang, W. *et al.* Genomics of drug sensitivity in cancer (gdsc): a resource for therapeutic biomarker discovery in cancer cells. *Nucleic acids research* **41**, D955–D961 (2012).

32. Liu, H. *et al.* Drugcombdb: a comprehensive database of drug combinations toward the discovery of combinatorial therapy. *Nucleic acids research* **48**, D871–D881 (2020).

33. Zagidullin, B. *et al.* Drugcomb: an integrative cancer drug combination data portal. *Nucleic acids research* **47**, W43–W51 (2019).

34. Barrett, T. *et al.* Ncbi geo: archive for functional genomics data sets—update. *Nucleic acids research* **41**, D991–D995 (2012).

35. LeCun, Y., Bengio, Y. & Hinton, G. Deep learning. *Nature* **521**, 436–444 (2015).

36. Deng, J. *et al.* A systematic study of key elements underlying molecular property prediction. *Nat. Commun.* **14**, 6395 (2023).

37. Preuer, K. *et al.* Deepsynergy: predicting anti-cancer drug synergy with deep learning. *Bioinformatics* **34**, 1538–1546 (2018).

38. LeCun, Y., Bottou, L., Bengio, Y. & Haffner, P. Gradient-based learning applied to document recognition. *Proc. IEEE* **86**, 2278–2324 (1998).

39. He, K., Zhang, X., Ren, S. & Sun, J. Spatial pyramid pooling in deep convolutional networks for visual recognition. *IEEE transactions on pattern analysis machine intelligence* **37**, 1904–1916 (2015).

40. Krizhevsky, A., Sutskever, I. & Hinton, G. E. Imagenet classification with deep convolutional neural networks. In *Proceedings of the 25th Annual Conference on Advances in neural information processing systems*, vol. 25 (2012).

41. Huang, G., Liu, Z., Van Der Maaten, L. & Weinberger, K. Q. Densely connected convolutional networks. In *Proceedings of the IEEE conference on computer vision and pattern recognition*, 4700–4708 (2017).

42. He, K., Zhang, X., Ren, S. & Sun, J. Deep residual learning for image recognition. In *Proceedings of the IEEE conference on computer vision and pattern recognition*, 770–778 (2016).

**43.** Kiranyaz, S. *et al.* 1d convolutional neural networks and applications: A survey. *Mech. systems signal processing* **151**, 107398 (2021).

**44.** Öztürk, H., Özgür, A. & Ozkirimli, E. Deepdta: deep drug–target binding affinity prediction. *Bioinformatics* **34**, i821–i829 (2018).

**45.** Zeng, Y., Chen, X., Luo, Y., Li, X. & Peng, D. Deep drug-target binding affinity prediction with multiple attention blocks. *Briefings bioinformatics* **22**, bbab117 (2021).

**46.** Rifaioglu, A. S. *et al.* Deepscreen: high performance drug–target interaction prediction with convolutional neural networks using 2-d structural compound representations. *Chem. science* **11**, 2531–2557 (2020).

**47.** Fernandez, M. *et al.* Toxic colors: the use of deep learning for predicting toxicity of compounds merely from their graphic images. *J. chemical information modeling* **58**, 1533–1543 (2018).

**48.** Zeng, X. *et al.* Accurate prediction of molecular properties and drug targets using a self-supervised image representation learning framework. *Nat. Mach. Intell.* **4**, 1004–1016 (2022).

**49.** Hochreiter, S. & Schmidhuber, J. Long short-term memory. *Neural computation* **9**, 1735–1780 (1997).

**50.** Chung, J., Gulcehre, C., Cho, K. & Bengio, Y. Empirical evaluation of gated recurrent neural networks on sequence modeling. *arXiv preprint arXiv:1412.3555* (2014).

**51.** Schuster, M. & Paliwal, K. K. Bidirectional recurrent neural networks. *IEEE transactions on Signal Process.* **45**, 2673–2681 (1997).

**52.** Mayr, A. *et al.* Large-scale comparison of machine learning methods for drug target prediction on chembl. *Chem. science* **9**, 5441–5451 (2018).

**53.** Goh, G. B., Hodas, N. O., Siegel, C. & Vishnu, A. Smiles2vec: An interpretable general-purpose deep neural network for predicting chemical properties. *arXiv preprint arXiv:1712.02034* (2017).

**54.** Kotsias, P.-C. *et al.* Direct steering of de novo molecular generation with descriptor conditional recurrent neural networks. *Nat. Mach. Intell.* **2**, 254–265 (2020).

**55.** Popova, M., Isayev, O. & Tropsha, A. Deep reinforcement learning for de novo drug design. *Sci. advances* **4**, eaap7885 (2018).

**56.** Olivecrona, M., Blaschke, T., Engkvist, O. & Chen, H. Molecular de-novo design through deep reinforcement learning. *J. cheminformatics* **9**, 1–14 (2017).

**57.** Zhou, J. *et al.* Graph neural networks: A review of methods and applications. *AI open* **1**, 57–81 (2020).

**58.** Li, R., Wang, S., Zhu, F. & Huang, J. Adaptive graph convolutional neural networks. In *Proceedings of the AAAI conference on artificial intelligence*, vol. 32 (2018).

**59.** Ma, Y. & Tang, J. *Deep learning on graphs* (Cambridge University Press, 2021).

**60.** Bruna, J., Zaremba, W., Szlam, A. & LeCun, Y. Spectral networks and locally connected networks on graphs. In *Proceedings of the 2nd International Conference on Learning Representations* (2014).

**61.** Defferrard, M., Bresson, X. & Vandergheynst, P. Convolutional neural networks on graphs with fast localized spectral filtering. *Adv. neural information processing systems* **29** (2016).

**62.** Kipf, T. N. & Welling, M. Semi-supervised classification with graph convolutional networks. In *Proceedings of the 5th International Conference on Learning Representations* (2017).

**63.** Gilmer, J., Schoenholz, S. S., Riley, P. F., Vinyals, O. & Dahl, G. E. Neural message passing for quantum chemistry. In *Proceedings of the International conference on machine learning*, 1263–1272 (2017).

**64.** Hamilton, W., Ying, Z. & Leskovec, J. Inductive representation learning on large graphs. In *Proceedings of the 30th Annual Conference on Advances in neural information processing systems*, 1024–1034 (2017).

**65.** Veličković, P. *et al.* Graph attention networks. In *Proceedings of the 6th International Conference on Learning Representations* (2018).

**66.** Xu, K., Hu, W., Leskovec, J. & Jegelka, S. How powerful are graph neural networks? In *Proceedings of the 7th International Conference on Learning Representations* (2019).

**67.** Vaswani, A. *et al.* Attention is all you need. In *Proceedings of the 30th Annual Conference on Advances in neural information processing systems*, 5998–6008 (2017).

**68.** Bo, D. *et al.* A survey on spectral graph neural networks. *arXiv preprint arXiv:2302.05631* (2023).

**69.** Rusch, T. K., Bronstein, M. M. & Mishra, S. A survey on oversmoothing in graph neural networks. *arXiv preprint arXiv:2303.10993* (2023).

**70.** Zhao, L. & Akoglu, L. Pairnorm: Tackling oversmoothing in gnns. *arXiv preprint arXiv:1909.12223* (2019).

**71.** Zhou, K. *et al.* Understanding and resolving performance degradation in deep graph convolutional networks. In *Proceedings of the 30th ACM international conference on information & knowledge management*, 2728–2737 (2021).

**72.** Ying, R. *et al.* Graph convolutional neural networks for web-scale recommender systems. In *Proceedings of the 24th ACM SIGKDD international conference on knowledge discovery & data mining*, 974–983 (2018).

**73.** Li, G., Muller, M., Thabet, A. & Ghanem, B. Deepgcns: Can gcns go as deep as cnns? In *Proceedings of the IEEE/CVF international conference on computer vision*, 9267–9276 (2019).

**74.** Han, J. *et al.* A survey of geometric graph neural networks: Data structures, models and applications. *Front. Comput. Sci.* **19**, 1911375 (2025).

**75.** Schütt, K. T., Sauceda, H. E., Kindermans, P.-J., Tkatchenko, A. & Müller, K.-R. Schnet–a deep learning architecture for molecules and materials. *The J. chemical physics* **148** (2018).

**76.** Schütt, K. T., Arbabzadah, F., Chmiela, S., Müller, K. R. & Tkatchenko, A. Quantum-chemical insights from deep tensor neural networks. *Nat. communications* **8**, 13890 (2017).

**77.** Satorras, V. G., Hoogeboom, E. & Welling, M. E (n) equivariant graph neural networks. In *International conference on machine learning*, 9323–9332 (PMLR, 2021).

**78.** Schütt, K., Unke, O. & Gastegger, M. Equivariant message passing for the prediction of tensorial properties and molecular spectra. In *International conference on machine learning*, 9377–9388 (PMLR, 2021).

**79.** Zhang, C., Song, D., Huang, C., Swami, A. & Chawla, N. V. Heterogeneous graph neural network. In *Proceedings of the 25th ACM SIGKDD international conference on knowledge discovery & data mining*, 793–803 (2019).

**80.** Wang, X. *et al.* Heterogeneous graph attention network. In *The world wide web conference*, 2022–2032 (2019).

**81.** Hu, Z., Dong, Y., Wang, K. & Sun, Y. Heterogeneous graph transformer. In *Proceedings of the web conference 2020*, 2704–2710 (2020).

**82.** Li, Z., Tu, X., Chen, Y. & Lin, W. Hetddi: a pre-trained heterogeneous graph neural network model for drug–drug interaction prediction. *Briefings Bioinforma.* **24**, bbad385 (2023).

**83.** Zhang, R. *et al.* H2d: Hierarchical heterogeneous graph learning framework for drug-drug interaction prediction. In *Proceedings of the 33rd ACM International Conference on Information and Knowledge Management*, 4283–4287 (2024).

**84.** Yang, K. *et al.* Analyzing learned molecular representations for property prediction. *J. chemical information modeling* **59**, 3370–3388 (2019).

**85.** Bento, A. P. *et al.* An open source chemical structure curation pipeline using rdkit. *J. Cheminformatics* **12**, 1–16 (2020).

**86.** Xiong, Z. *et al.* Pushing the boundaries of molecular representation for drug discovery with the graph attention mechanism. *J. medicinal chemistry* **63**, 8749–8760 (2019).

**87.** Withnall, M., Lindelöf, E., Engkvist, O. & Chen, H. Building attention and edge message passing neural networks for bioactivity and physical–chemical property prediction. *J. cheminformatics* **12**, 1–18 (2020).

**88.** Fang, X. *et al.* Geometry-enhanced molecular representation learning for property prediction. *Nat. Mach. Intell.* **4**, 127–134 (2022).

**89.** You, J., Liu, B., Ying, Z., Pande, V. & Leskovec, J. Graph convolutional policy network for goal-directed molecular graph generation. *Adv. neural information processing systems* **31** (2018).

**90.** Zhou, Z., Kearnes, S., Li, L., Zare, R. N. & Riley, P. Optimization of molecules via deep reinforcement learning. *Sci. reports* **9**, 10752 (2019).

**91.** Khemchandani, Y. *et al.* Deepgraphmolgen, a multi-objective, computational strategy for generating molecules with desirable properties: a graph convolution and reinforcement learning approach. *J. cheminformatics* **12**, 1–17 (2020).

**92.** Xu, C., Liu, Q., Huang, M. & Jiang, T. Reinforced molecular optimization with neighborhood-controlled grammars. In *Proceedings of the 33th Annual Conference on Advances in Neural Information Processing Systems*, 8366–8377 (2020).

**93.** Ba, J. L., Kiros, J. R. & Hinton, G. E. Layer normalization. *arXiv preprint arXiv:1607.06450* (2016).

**94.** Devlin, J., Chang, M.-W., Lee, K. & Toutanova, K. BERT: pre-training of deep bidirectional transformers for language understanding. In *Proceedings of Conference of the North American Chapter of the Association for Computational Linguistics*, 4171–4186 (2019).

**95.** Shaw, P., Uszkoreit, J. & Vaswani, A. Self-attention with relative position representations. In *Proceedings of the 2018 Conference of the North American Chapter of the Association for Computational Linguistics: Human Language Technologies*, 464–468 (2018).

**96.** Su, J. *et al.* Roformer: Enhanced transformer with rotary position embedding. *Neurocomputing* **568**, 127063 (2024).

**97.** Dosovitskiy, A. *et al.* An image is worth 16x16 words: Transformers for image recognition at scale. In *Proceedings of the 9th International Conference on Learning Representations* (2021).

**98.** Ying, C. *et al.* Do transformers really perform badly for graph representation? In *Proceedings of the 34th Annual Conference on Advances in Neural Information Processing Systems*, vol. 34, 28877–28888 (2021).

**99.** Lin, X. V., Socher, R. & Xiong, C. Multi-hop knowledge graph reasoning with reward shaping. In *Proceedings of the 2018 Conference on Empirical Methods in Natural Language Processing*, 3243–3253 (2018).

**100.** Chen, T., Kornblith, S., Norouzi, M. & Hinton, G. A simple framework for contrastive learning of visual representations. In *Proceedings of the International conference on machine learning*, 1597–1607 (2020).

**101.** You, Y. *et al.* Graph contrastive learning with augmentations. In *Proceedings of the 34th Annual Conference on Advances in neural information processing systems*, vol. 33, 5812–5823 (2020).

**102.** Qiu, J. *et al.* Gcc: Graph contrastive coding for graph neural network pre-training. In *Proceedings of the 26th ACM SIGKDD international conference on knowledge discovery & data mining*, 1150–1160 (2020).

**103.** Wu, L., Lin, H., Tan, C., Gao, Z. & Li, S. Z. Self-supervised learning on graphs: Contrastive, generative, or predictive. *IEEE Transactions on Knowl. Data Eng.* (2021).

**104.** Donsker, M. D. & Varadhan, S. S. Asymptotic evaluation of certain markov process expectations for large time. iv. *Commun. on pure applied mathematics* **36**, 183–212 (1983).

**105.** Goodfellow, I. *et al.* Generative adversarial nets. In *Proceedings of the 27th Annual Conference on Advances in neural information processing systems*, 5998–6008 (2014).

**106.** Gutmann, M. & Hyvärinen, A. Noise-contrastive estimation: A new estimation principle for unnormalized statistical models. In *Proceedings of the thirteenth international conference on artificial intelligence and statistics*, 297–304 (2010).

**107.** Zhao, C., Liu, S., Huang, F., Liu, S. & Zhang, W. Csgnn: Contrastive self-supervised graph neural network for molecular interaction prediction. In *Proceedings of the IJCAI*, 3756–3763 (2021).

**108.** Wang, Y., Wang, J., Cao, Z. & Barati Farimani, A. Molecular contrastive learning of representations via graph neural networks. *Nat. Mach. Intell.* **4**, 279–287 (2022).

**109.** Wang, T. & Isola, P. Understanding contrastive representation learning through alignment and uniformity on the hypersphere. In *Proceedings of the International conference on machine learning*, 9929–9939 (PMLR, 2020).

**110.** Fang, Y. *et al.* Molecular contrastive learning with chemical element knowledge graph. In *Proceedings of the AAAI conference on artificial intelligence*, vol. 36, 3968–3976 (2022).

**111.** Li, H. *et al.* A knowledge-guided pre-training framework for improving molecular representation learning. *Nat. Commun.* **14**, 7568 (2023).

**112.** Sun, M., Xing, J., Wang, H., Chen, B. & Zhou, J. Mocl: data-driven molecular fingerprint via knowledge-aware contrastive learning from molecular graph. In *Proceedings of the 27th ACM SIGKDD conference on knowledge discovery & data mining*, 3585–3594 (2021).

**113.** Fang, Y. *et al.* Knowledge-aware contrastive molecular graph learning. *arXiv preprint arXiv:2103.13047* (2021).

**114.** Fang, Y. *et al.* Knowledge graph-enhanced molecular contrastive learning with functional prompt. *Nat. Mach. Intell.* **5**, 542–553 (2023).

**115.** Liu, Y. *et al.* Roberta: A robustly optimized bert pretraining approach. *arXiv preprint arXiv:1907.11692* (2019).

**116.** Raffel, C. *et al.* Exploring the limits of transfer learning with a unified text-to-text transformer. *The J. Mach. Learn. Res.* **21**, 5485–5551 (2020).

**117.** Joshi, M. *et al.* Spanbert: Improving pre-training by representing and predicting spans. *Transactions association for computational linguistics* **8**, 64–77 (2020).

118. He, K. *et al.* Masked autoencoders are scalable vision learners. In *Proceedings of the IEEE/CVF conference on computer vision and pattern recognition*, 16000–16009 (2022).

119. Bao, H., Dong, L., Piao, S. & Wei, F. Beit: BERT pre-training of image transformers. In *Proceedings of the Tenth International Conference on Learning Representations* (2022).

120. Wang, S., Guo, Y., Wang, Y., Sun, H. & Huang, J. Smiles-bert: large scale unsupervised pre-training for molecular property prediction. In *Proceedings of the 10th ACM international conference on bioinformatics, computational biology and health informatics*, 429–436 (2019).

121. Chithrananda, S., Grand, G. & Ramsundar, B. Chemberta: large-scale self-supervised pretraining for molecular property prediction. *arXiv preprint arXiv:2010.09885* (2020).

122. Ross, J. *et al.* Large-scale chemical language representations capture molecular structure and properties. *Nat. Mach. Intell.* **4**, 1256–1264 (2022).

123. Hu, W. *et al.* Strategies for pre-training graph neural networks. In *Proceedings of the 8th International Conference on Learning Representations* (2020).

124. Rong, Y. *et al.* Self-supervised graph transformer on large-scale molecular data. In *Proceedings of the 33th Conference on Neural Information Processing Systems* (2020).

125. Brown, T. *et al.* Language models are few-shot learners. In *Proceedings of the 33th Annual Conference on Advances in neural information processing systems*, 1877–1901 (2020).

126. Dai, Z. *et al.* Transformer-xl: Attentive language models beyond a fixed-length context. In *Proceedings of the 57th Conference of the Association for Computational Linguistics*, 2978–2988 (2019).

127. Chowdhery, A. *et al.* Palm: Scaling language modeling with pathways. *J. Mach. Learn. Res.* **24**, 1–113 (2023).

128. Zhu, J., Liu, Y., Zhang, Y., Chen, Z. & Wu, X. Multi-attribute discriminative representation learning for prediction of adverse drug-drug interaction. *IEEE transactions on pattern analysis machine intelligence* **44**, 10129–10144 (2021).

129. Veličković, P. *et al.* Deep graph infomax. In *Proceedings of the 7th International Conference on Learning Representations* (2019).

130. Sun, F.-Y., Hoffmann, J., Verma, V. & Tang, J. Infograph: Unsupervised and semi-supervised graph-level representation learning via mutual information maximization. In *Proceedings of the 8th International Conference on Learning Representations* (2020).

131. Radford, A. *et al.* Learning transferable visual models from natural language supervision. In *Proceedings of the International conference on machine learning*, 8748–8763 (PMLR, 2021).

132. Bao, H. *et al.* Vlmo: Unified vision-language pre-training with mixture-of-modality-experts. In *Proceedings of the 35th Annual Conference on Advances in Neural Information Processing Systems*, 32897–32912 (2022).

133. Li, J., Li, D., Xiong, C. & Hoi, S. Blip: Bootstrapping language-image pre-training for unified vision-language understanding and generation. In *Proceedings of the International conference on machine learning*, 12888–12900 (PMLR, 2022).

134. Pinheiro, G. A., Da Silva, J. L. & Quiles, M. G. Smiclr: contrastive learning on multiple molecular representations for semisupervised and unsupervised representation learning. *J. Chem. Inf. Model.* **62**, 3948–3960 (2022).

135. Zhu, Y. *et al.* Molecular contrastive pretraining with collaborative featurizations. *J. Chem. Inf. Model.* **64**, 1112–1122 (2024).

136. Stärk, H. *et al.* 3d infomax improves gnns for molecular property prediction. In *Proceedings of the International Conference on Machine Learning*, 20479–20502 (2022).

137. Li, S., Zhou, J., Xu, T., Dou, D. & Xiong, H. Geomgcl: Geometric graph contrastive learning for molecular property prediction. In *Proceedings of the AAAI conference on artificial intelligence*, 4541–4549 (2022).

138. Liu, S. *et al.* Multi-modal molecule structure–text model for text-based retrieval and editing. *Nat. Mach. Intell.* **5**, 1447–1457 (2023).

139. Edwards, C., Zhai, C. & Ji, H. Text2mol: Cross-modal molecule retrieval with natural language queries. In *Proceedings of the 2021 Conference on Empirical Methods in Natural Language Processing*, 595–607 (2021).

140. Zong, Y., Mac Aodha, O. & Hospedales, T. Self-supervised multimodal learning: A survey. *IEEE Transactions on Pattern Analysis Mach. Intell.* (2024).

**141.** Chen, Y.-C. *et al.* Uniter: Universal image-text representation learning. In *Proceedings of the European conference on computer vision*, 104–120 (Springer, 2020).

**142.** Kim, W., Son, B. & Kim, I. Vilt: Vision-and-language transformer without convolution or region supervision. In *Proceedings of the International conference on machine learning*, 5583–5594 (2021).

**143.** Li, J. *et al.* Align before fuse: Vision and language representation learning with momentum distillation. In *Proceedings of the 34th Annual Conference on Advances in neural information processing systems*, 9694–9705 (2021).

**144.** Zhang, X. *et al.* Dual-view learning based on images and sequences for molecular property prediction. *IEEE J. Biomed. Heal. Informatics* (2023).

**145.** Liu, Z. *et al.* Molca: Molecular graph-language modeling with cross-modal projector and uni-modal adapter. In *Proceedings of the Conference on Empirical Methods in Natural Language Processing*, 15623–15638 (Association for Computational Linguistics, 2023).

**146.** Zeng, Z., Yao, Y., Liu, Z. & Sun, M. A deep-learning system bridging molecule structure and biomedical text with comprehension comparable to human professionals. *Nat. communications* **13**, 862 (2022).

**147.** Pei, Q. *et al.* BioT5: Enriching cross-modal integration in biology with chemical knowledge and natural language associations. In *Proceedings of the 2023 Conference on Empirical Methods in Natural Language Processing*, 1102–1123 (2023).

**148.** Perozzi, B., Al-Rfou, R. & Skiena, S. Deepwalk: online learning of social representations. In *Proceedings of the 20th ACM SIGKDD International Conference on Knowledge Discovery and Data Mining*, 701–710 (2014).

**149.** Wang, X. *et al.* Deepr2cov: deep representation learning on heterogeneous drug networks to discover anti-inflammatory agents for covid-19. *Briefings bioinformatics* **22**, bbab226 (2021).

**150.** Wang, X. *et al.* Bioerp: biomedical heterogeneous network-based self-supervised representation learning approach for entity relationship predictions. *Bioinformatics* **37**, 4793–4800 (2021).

**151.** Liu, Z. *et al.* Molxpt: Wrapping molecules with text for generative pre-training. In *Proceedings of the 61st Annual Meeting of the Association for Computational Linguistics*, 1606–1616 (2023).

**152.** Zhang, Z., Liu, Q., Wang, H., Lu, C. & Lee, C.-K. Motif-based graph self-supervised learning for molecular property prediction. In *Proceedings of the 34th Annual Conference on Advances in Neural Information Processing Systems*, 15870–15882 (2021).

**153.** Bagal, V., Aggarwal, R., Vinod, P. & Priyakumar, U. D. Molgpt: molecular generation using a transformer-decoder model. *J. Chem. Inf. Model.* **62**, 2064–2076 (2021).

**154.** Liu, Y. *et al.* Graph self-supervised learning: A survey. *IEEE Transactions on Knowl. Data Eng.* **35**, 5879–5900 (2022).

**155.** Xiong, Z. *et al.* Multi-relational contrastive learning graph neural network for drug-drug interaction event prediction. In *Proceedings of the AAAI Conference on Artificial Intelligence*, 5339–5347 (2023).

**156.** Su, X., Hu, P., You, Z.-H., Philip, S. Y. & Hu, L. Dual-channel learning framework for drug-drug interaction prediction via relation-aware heterogeneous graph transformer. In *Proceedings of the AAAI Conference on Artificial Intelligence*, 1, 249–256 (2024).

**157.** Wang, Y., Min, Y., Chen, X. & Wu, J. Multi-view graph contrastive representation learning for drug-drug interaction prediction. In *Proceedings of the 34th The web conference*, 2921–2933 (2021).

**158.** Han, Z., Gao, C., Liu, J., Zhang, J. & Zhang, S. Q. Parameter-efficient fine-tuning for large models: A comprehensive survey. *arXiv preprint arXiv:2403.14608* (2024).

**159.** Xu, L., Xie, H., Qin, S.-Z. J., Tao, X. & Wang, F. L. Parameter-efficient fine-tuning methods for pretrained language models: A critical review and assessment. *arXiv preprint arXiv:2312.12148* (2023).

**160.** Houlsby, N. *et al.* Parameter-efficient transfer learning for nlp. In *International conference on machine learning*, 2790–2799 (2019).

**161.** Li, X. L. & Liang, P. Prefix-tuning: Optimizing continuous prompts for generation. In *Proceedings of the 59th Annual Meeting of the Association for Computational Linguistics*, 4582–4597 (2021).

**162.** Lester, B., Al-Rfou, R. & Constant, N. The power of scale for parameter-efficient prompt tuning. In *Proceedings of the Conference on Empirical Methods in Natural Language Processing*, 3045–3059 (2021).

**163.** Zaken, E. B., Goldberg, Y. & Ravfogel, S. Bitfit: Simple parameter-efficient fine-tuning for transformer-based masked language-models. In *Proceedings of the 60th Annual Meeting of the Association for Computational Linguistics*, 1–9 (2022).

**164.** Hu, E. J. *et al.* Lora: Low-rank adaptation of large language models. In *Proceedings of the Tenth International Conference on Learning Representations* (2022).

**165.** Wu, K. *et al.* Tamgen: drug design with target-aware molecule generation through a chemical language model. *Nat. Commun.* **15**, 9360 (2024).

**166.** Feng, W. *et al.* Generation of 3d molecules in pockets via a language model. *Nat. Mach. Intell.* **6**, 62–73 (2024).

**167.** Wang, J. *et al.* 3dsmiles-gpt: 3d molecular pocket-based generation with token-only large language model. *Chem. Sci.* **16**, 637–648 (2025).

**168.** Edwards, C. *et al.* Translation between molecules and natural language. In *Proceedings of the Conference on Empirical Methods in Natural Language Processing*, 375–413 (2022).

**169.** Chen, Y. *et al.* From artificially real to real: Leveraging pseudo data from large language models for low-resource molecule discovery. In *Proceedings of the AAAI Conference on Artificial Intelligence*, 21958–21966 (2024).

**170.** Zeng, Z. *et al.* Chatmol: interactive molecular discovery with natural language. *Bioinformatics* **40**, btae534 (2024).

**171.** Christofidellis, D. *et al.* Unifying molecular and textual representations via multi-task language modelling. In *Proceedings of the International Conference on Machine Learning*, 6140–6157 (2023).

**172.** Livne, M. *et al.* nach0: Multimodal natural and chemical languages foundation model. *Chem. Sci.* **15**, 8380–8389 (2024).

**173.** Liu, P., Ren, Y., Tao, J. & Ren, Z. Git-mol: A multi-modal large language model for molecular science with graph, image, and text. *Comput. biology medicine* **171**, 108073 (2024).

**174.** Beltagy, I., Lo, K. & Cohan, A. Scibert: A pretrained language model for scientific text. In *Proceedings of the Conference on Empirical Methods in Natural Language Processing and the 9th International Joint Conference on Natural Language Processing*, 3613–3618 (2019).

**175.** Liu, Z. *et al.* Swin transformer: Hierarchical vision transformer using shifted windows. In *Proceedings of the IEEE/CVF international conference on computer vision*, 10012–10022 (2021).

**176.** Guo, Z., Sharma, P. K., Martinez, A., Du, L. & Abraham, R. Multilingual molecular representation learning via contrastive pre-training. In *Proceedings of the 60th Annual Meeting of the Association for Computational Linguistics*, 3441–3453 (2022).

**177.** Zhu, J. *et al.* Dual-view molecular pre-training. In *Proceedings of the 29th ACM SIGKDD Conference on Knowledge Discovery and Data Mining*, 3615–3627 (2023).

**178.** Liu, S. *et al.* Pre-training molecular graph representation with 3d geometry. In *Proceedings of the International Conference on Learning Representations* (2022).

**179.** Schütt, K. *et al.* Schnet: A continuous-filter convolutional neural network for modeling quantum interactions. *Adv. neural information processing systems* **30** (2017).

**180.** Zhu, J. *et al.* Unified 2d and 3d pre-training of molecular representations. In *Proceedings of the 28th ACM SIGKDD Conference on Knowledge Discovery and Data Mining*, 2626–2636 (2022).

**181.** Zang, X., Zhang, J. & Tang, B. Self-supervised pre-training via multi-view graph information bottleneck for molecular property prediction. *IEEE J. Biomed. Heal. Informatics* (2024).

**182.** Yu, Q. *et al.* Multimodal molecular pretraining via modality blending. In *Proceedings of the 12th International Conference on Learning Representations* (2024).

**183.** Tang, X., Tran, A., Tan, J. & Gerstein, M. B. Mollm: a unified language model for integrating biomedical text with 2d and 3d molecular representations. *Bioinformatics* **40**, i357–i368 (2024).

**184.** Irwin, R., Dimitriadis, S., He, J. & Bjerrum, E. J. Chemformer: a pre-trained transformer for computational chemistry. *Mach. Learn. Sci. Technol.* **3**, 015022 (2022).

**185.** Liu, S., Demirel, M. F. & Liang, Y. N-gram graph: Simple unsupervised representation for graphs, with applications to molecules. In *Proceedings of the 32th Annual Conference on Advances in neural information processing systems*, 8464–8476 (2019).

**186.** Su, B. *et al.* A molecular multimodal foundation model associating molecule graphs with natural language. *arXiv preprint arXiv:2209.05481* (2022).

**187.** Ye, Y. *et al.* Hierarchical structure-aware graph prompting for drug-drug interaction prediction. In *Proceedings of the Joint European Conference on Machine Learning and Knowledge Discovery in Databases*, 36–54 (2024).

**188.** Ashburn, T. T. & Thor, K. B. Drug repositioning: identifying and developing new uses for existing drugs. *Nat. reviews Drug discovery* **3**, 673–683 (2004).

**189.** Wang, J., Xiao, Y., Shang, X. & Peng, J. Predicting drug–target binding affinity with cross-scale graph contrastive learning. *Briefings Bioinforma.* **25**, bbad516 (2024).

**190.** Luo, Z., Wu, W., Sun, Q. & Wang, J. Accurate and transferable drug–target interaction prediction with druglamp. *Bioinformatics* **40**, btae693 (2024).

**191.** Krenn, M., Häse, F., Nigam, A., Friederich, P. & Aspuru-Guzik, A. Self-referencing embedded strings (selfies): A 100% robust molecular string representation. *Mach. Learn. Sci. Technol.* **1**, 045024 (2020).

**192.** Li, J. *et al.* Empowering molecule discovery for molecule-caption translation with large language models: A chatgpt perspective. *IEEE Transactions on Knowl. Data Eng.* (2024).

**193.** Liu, Z. *et al.* Molca: Molecular graph-language modeling with cross-modal projector and uni-modal adapter. In *Proceedings of the 2023 Conference on Empirical Methods in Natural Language Processing*, 15623–15638 (2023).

**194.** Taylor, R. *et al.* Galactica: A large language model for science. *arXiv preprint arXiv:2211.09085* (2022).

**195.** Li, S. *et al.* Towards 3d molecule-text interpretation in language models. In *Proceedings of the 12th International Conference on Learning Representations* (2024).

**196.** Touvron, H. *et al.* Llama 2: Open foundation and fine-tuned chat models. *arXiv preprint arXiv:2307.09288* (2023).

**197.** Luo, Y., Yang, K., Hong, M., Liu, X. Y. & Nie, Z. Molfm: A multimodal molecular foundation model. *arXiv preprint arXiv:2307.09484* (2023).

**198.** Bordes, A., Usunier, N., Garcia-Duran, A., Weston, J. & Yakhnenko, O. Translating embeddings for modeling multi-relational data. *Proc. 27th Annu. Conf. on Adv. neural information processing systems* 2787–2795 (2013).

**199.** Zhang, J., Bian, Y., Chen, Y. & Yao, Q. Unimot: Unified molecule-text language model with discrete token representation. *arXiv preprint arXiv:2408.00863* (2024).

**200.** Li, J., Li, D., Savarese, S. & Hoi, S. Blip-2: Bootstrapping language-image pre-training with frozen image encoders and large language models. In *Proceedings of the International conference on machine learning*, 19730–19742 (2023).
